# The Neurod1/4-Ntrk3-Src pathway regulates gonadotrope cell adhesion and motility

**DOI:** 10.1038/s41420-023-01615-7

**Published:** 2023-09-01

**Authors:** Charles Le Ciclé, Vincent Pacini, Nicolas Rama, Servane Tauszig-Delamasure, Eloïse Airaud, Florence Petit, Simon de Beco, Joëlle Cohen-Tannoudji, David L’hôte

**Affiliations:** 1 Université Paris Cité, CNRS, Inserm, Unité de Biologie Fonctionnelle et Adaptative, F-75013 Paris, France; 2 Centre de Recherche en Cancérologie de Lyon, Inserm U1052, CNRS UMR 5286, Centre Léon Bérard, Université Lyon1, 69008 Lyon, France; 3 Institut NeuroMyoGène - CNRS UMR 5310 - Inserm U1217 de Lyon - UCBL Lyon 1, Faculté de Médecine et de Pharmacie, Lyon, France; 4 Present Address: Université Paris Cité, CNRS, Institut Jacques Monod, F-75013 Paris, France; 5 Present Address: Faculty of Pharmacy, Université de Montréal, Montréal, QC H3T 1J4 Canada

**Keywords:** Differentiation, Focal adhesion, Reproductive biology

## Abstract

Pituitary gonadotrope cells are essential for the endocrine regulation of reproduction in vertebrates. These cells emerge early during embryogenesis, colonize the pituitary glands and organize in tridimensional networks, which are believed to be crucial to ensure proper regulation of fertility. However, the molecular mechanisms regulating the organization of gonadotrope cell population during embryogenesis remain poorly understood. In this work, we characterized the target genes of NEUROD1 and NEUROD4 transcription factors in the immature gonadotrope αT3-1 cell model by in silico functional genomic analyses. We demonstrated that NEUROD1/4 regulate genes belonging to the focal adhesion pathway. Using CRISPR/Cas9 knock-out approaches, we established a double NEUROD1/4 knock-out αT3-1 cell model and demonstrated that NEUROD1/4 regulate cell adhesion and cell motility. We then characterized, by immuno-fluorescence, focal adhesion number and signaling in the context of NEUROD1/4 insufficiency. We demonstrated that NEUROD1/4 knock-out leads to an increase in the number of focal adhesions associated with signaling abnormalities implicating the c-Src kinase. We further showed that the neurotrophin tyrosine kinase receptor 3 NTRK3, a target of NEUROD1/4, interacts physically with c-Src. Furthermore, using motility rescue experiments and time-lapse video microscopy, we demonstrated that NTRK3 is a major regulator of gonadotrope cell motility. Finally, using a *Ntrk3* knock-out mouse model, we showed that NTRK3 regulates gonadotrope cells positioning in the developing pituitary, in vivo. Altogether our study demonstrates that the Neurod1/4-Ntrk3-cSrc pathway is a major actor of gonadotrope cell mobility, and thus provides new insights in the regulation of gonadotrope cell organization within the pituitary gland.

## Introduction

In multi-cellular organisms, the basic helix-loop-helix (bHLH) super-family regroups highly conserved transcription factors, often implicated in critical developmental processes. Amongst them, the Neurod/g sub-family emerged as major regulator of neurogenesis [1], playing critical roles in neural cell fate differentiation, but also in neuron proliferation, survival, and migration. This pro-neural sub-family is composed of four *Neurod* members: *Neurod1*, *Neurod2*, *Neurod4*, and *Neurod6* and three Neurogenin, *Neurog1, Neurog2,* and *Neurog3*. NEUROD1 (ND1) and NEUROD4 (ND4) along with Neurogenin factors are able to reprogram progenitor cells toward a neural fate whereas ND2 and ND6 are implicated in neuronal identity maintenance (for review [2]). These factors can functionally compensate each other, displaying partially overlapping expression patterns and sharing a highly conserved DNA binding domain [3]. Neurod/g are also involved in specification of non-neural cells such as retinal photoreceptors [4], amacrine cells [5], pancreatic endocrine β cells [6], and entero-endocrine cells [7]. Furthermore, ND1 and ND4 are known to be expressed during pituitary gland development [8–10].

The pituitary gland is a common feature of all vertebrates. The endocrine part of this gland contains six endocrine lineages in the mouse: somatotrope, lactotrope, thyrotrope, corticotrope, melanotrope and gonadotrope. Each of them secretes specific hormones implicated in the homeostatic regulation of important physiological functions. For example, the gonadotrope cells secrete the gonadotropins, LH and FSH, regulating gonadal steroidogenesis and gametogenesis and thus, fine-tuning the reproductive function. This gland differentiates from a cranial placode, at day 8.5 of embryonic development (E8.5) in the mouse [11]. Cranial placodes are tissular hotspots of cell division and morphogenesis, originating at the boundary between neural and non-neural ectoderms [12]. They are known to produce various types of migratory cells such as neural and, as more recently demonstrated, endocrine cells [13]. Interestingly, all cranial placodes are neurogenic except for anterior pituitary and lens ones, suggesting an ontogenetic proximity between neural and pituitary development. Early during embryogenesis (E9.5), the pituitary placode gives rise to the Rathke’s pouch, the pituitary anlage. Progenitor cells proliferate and emigrate from this pouch to give birth to the nascent pituitary gland (around E12.5) [14]. From E13.5, under morphogenetic cues, the progenitors then differentiate progressively into the different endocrine lineages [15]. During this maturation, most of the pituitary lineages, and especially the gonadotrope one, are known to interconnect into cell networks [16], suggesting that cell motility might be important to establish the complex tridimensional organization of the embryonic pituitary.

Interestingly, pituitary differentiation requires several pro-neural transcription factors, highlighting similarities between pituitary and neural development. For example, as previously mentioned, the pro-neural transcription factors ND1 and ND4 are expressed in the developing pituitary [8–10]. In agreement with these expression patterns, *Neurod1* or *Neurdo4* knock-out leads to mild differentiation defects of corticotropes [17], lactotropes, and somatotropes, respectively [10]. While pituitary differentiation has not been investigated in *Neurdo1/4* double mutant, differentiation defects of the somatotrope, corticotrope and gonadotrope lineages have been observed in a triple knock-out with *Ascl1*, coding a closely related bHLH transcription factor [18], demonstrating the importance of these factors for pituitary endocrine lineage maturation. However, the roles of ND1 and ND4, especially during gonadotrope specification, remains poorly characterized. In this work, we have studied ND1/4 molecular function during gonadotrope cell differentiation using a classical set of three immortalized mouse cell lines recapitulating three major steps of the gonadotrope differentiation process: the αT1-1 cell line, a model of early progenitors, the αT3-1 cells, exhibiting molecular signature of immature gonadotropes and finally the LβT2 cell line, representative of mature gonadotrope cells [19]. We demonstrate here that (i) ND1 and ND4 are critical factors during gonadotrope differentiation, regulating cell motility through a Ntrk3/c-Src pathway and (ii) NTRK3 tyrosine kinase receptor is crucial for the organization of the gonadotrope cell population during pituitary organogenesis.

## Results

### *Neurod1* and *Neurod4* are differentially expressed in a cellular model of gonadotrope differentiation

**Fig. 1:**
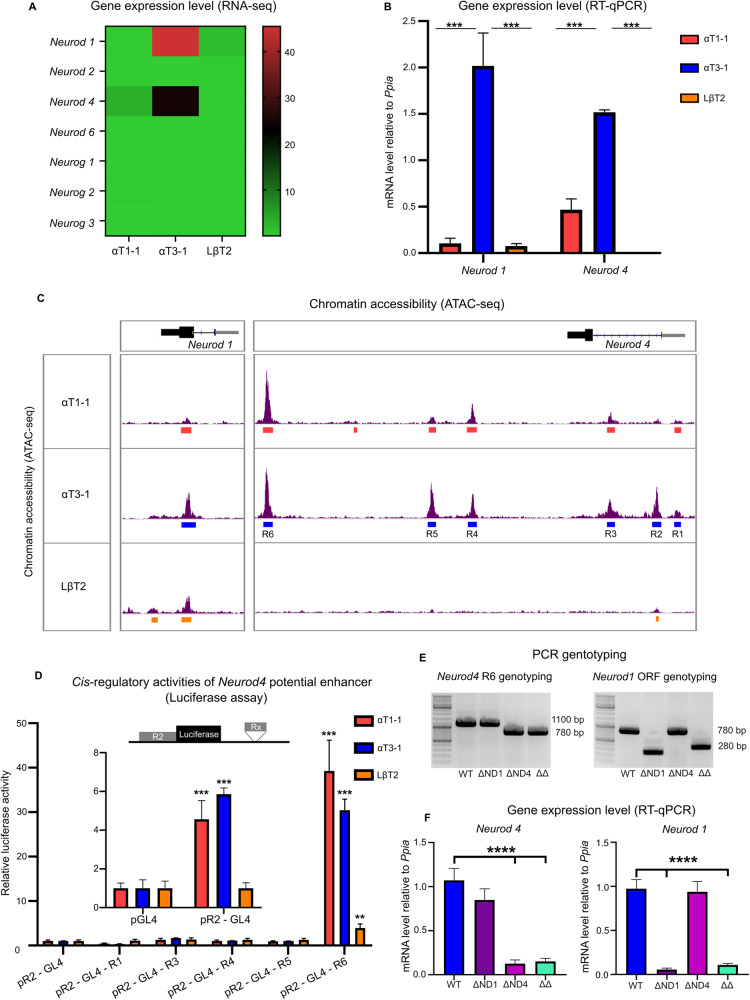
Characterization of *Neurod/g* gene expression in a model of gonadotrope cell differentiation. **A** Heatmap of *Neurod/g* gene expression in αT1-1, αT3-1, and LβT2 cells according to published RNA-seq (GSE104513). Color scale represents the normalized expression values. **B** Expression of *Neurod1* and *Neurod4* in αT1-1, αT3-1, and LβT2 cells. Gene expression levels were measured by RT-qPCR and normalized to *Ppia* (Cyclophilin A). Data are the normalized mean ± SEM of four independent experiments. *Neurod1* and *Neurod4* transcript levels were compared between the three cell lines by ANOVA followed by Tukey multiple comparison test. ***p < 0.001. **C** Chromatin accessibility of *Neurod1* and *Neurod4* loci in αT1-1, αT3-1, and LβT2 cells. Chromatin accessibility was investigated by ATAC-seq (assay for transposase-accessible chromatin with high-throughput sequencing) in the three gonadotrope cell lines. ATAC-seq tracks are shown for *Neurod1* (left part of the panel) and *Neurod4* (right part of the panel) loci. Gene exons are depicted as black boxes and proximal promoters are depicted as gray box in 5 prime of the ORFs. For each cell line, identified accessible chromatin regions are depicted as colored rectangles under each track. **D** *Cis*-regulatory activity of *Neurod4* potential enhancers in gonadotrope cells. αT1-1, αT3-1, and LβT2 cells were transiently transfected with a GL4 luciferase reporter cassette under the control of *Neurod4* promoter, here referred as R2 (pR2-GL4) or with this construct harboring potential cis-regulatory regions cloned in 3 prime of the *luciferase* cassette (pR2-GL4-Rx constructs, with x = R1 to R6 except R2). The activity of pR2-GL4 was normalized to the control pGL4 empty backbone activity (inset) and then pR2-GL4-Rx activities were normalized to pR2-GL4 activity. Relative luciferase activities are the normalized ratio between firefly luciferase over renilla luciferase. ANOVA followed by Dunnett’s multiple comparison test was performed independently for each cell line. Results are the mean ± SEM of six independent experiments. Significant difference with pR2-GL4 construct: **p < 0.01; ***p < 0.001. **E** Deletion of the genomic sequence of *Neurod4* R6 enhancer and *Neurod1* ORF was carried out in αT3-1 cells using CRISPR/Cas9. For *Neurod4*, genotyping PCR amplified a 1100 bp amplicon in WT cells while stable *Neurod4* mutant cells (ΔND4) carried a deletion of about 320 bp, removing the R6 enhancer (left part of the panel, 780 bp amplicon). For *Neurod1*, a 780 bp amplicon was detected in WT cells while stable *Neurod1* mutant cells (ΔND1) carried a deletion of about 500 bp, which encompassed *Neurod1* ATG first codon (right part of the panel, 280 bp amplicon). The double mutant αT3-1 cell line (ΔΔ) was generated by deleting R6 enhancer on the *Neurod1* mutant background. **F** For each genotype, homozygous clones were tested for *Neurod4* and *Neurod1* expression by RT-qPCR. Gene expression levels were normalized to *Ppia*. Data are the normalized mean ± SEM of four independent experiments. Gene expression levels of mutant clones were compared to WT by ANOVA followed by Dunnett’s multiple comparison test. ****p < 0.0001.

We first investigated the expression of *Neurod/g* genes in the αT1-1, αT3-1, and LβT2 cell lines. According to RNA-seq data [20], we found that *Neurod1* and *Neurod4* are the only *Neurod/g* expressed genes during gonadotrope differentiation, mostly in immature and to a lesser extent in progenitor gonadotrope cells (Fig. 1A). We confirmed this expression pattern by RT-qPCR assay (Fig. 1B). We then investigated whether *Neurod1* and *Neurod4* regulatory regions were differentially activated during gonadotrope differentiation using our published ATAC-seq data [21]. Only *Neurod1* proximal promoter displayed chromatin accessibility, mainly in immature gonadotrope cells (Fig. 1C, left part). In contrast, we found six accessible chromatin regions in *Neurod4* locus of αT1-1 and αT3-1 cells but not in LβT2 cells (Fig. 1C, right part), consistently with gene expression pattern. This indicates that *Neurod4* expression might strongly depends on several potential enhancers scattered within the locus. We then tested enhancer *cis-*regulatory activities using luciferase reporter assays. As expected, *Neurod4* promoter region (R2) was active specifically in αT1-1 and αT3-1 cells (Fig. 1D, insert). Among the potential enhancers, only R6 dramatically increased promoter basal activity in progenitor and immature gonadotrope cells (Fig. 1D). To further demonstrate that R6 is a functional *Neurod4* enhancer, we generated stable R6 deletion in αT3-1 cells using CRISPR/Cas9 (these cells are hereafter named ΔND4, Fig. 1E) and measured *Neurod4* expression by RT-qPCR. R6 deletion in immature gonadotropes induced a 90% decrease in *Neurod4* transcript levels (Fig. 1F), demonstrating that the R6 enhancer is necessary for *Neurod4* expression in αT3-1 cells. To our knowledge, this *Neurod4* enhancer had not been described yet. As this evolutionary conserved region displays chromatin accessibility in tissues such as brain and retina (according to the UCSC Genome Browser databases), it might also be a critical *Neurod4* regulatory region beyond the pituitary gland. The ΔND4 cells can be considered as an immature gonadotrope *Neurod4* knock-out cellular model, allowing the analysis of ND4 functions in this cell type.

To study ND1 roles in immature gonadotropes, we created a αT3-1 *Neurod1* mutant cell line by deleting the first ~500 bp of the *Neurod1* ORF with CRISPR/Cas9 (Fig. 1E, ΔND1 cells), which successfully abolished gene expression (Fig. 1F). Finally, in order to investigate potential functional compensations between the two NEUROD factors, we established a double mutant αT3-1 cell line by deleting *Neurod4*-R6 in ΔND1 cells (Fig. 1E, ΔΔ cells) leading to a strong down-expression of both *Neurod1* and *Neurod4* (Fig. 1F).

### Characterization of NEUROD1 and NEUROD4 potential regulome in immature gonadotrope cells

**Fig. 2:**
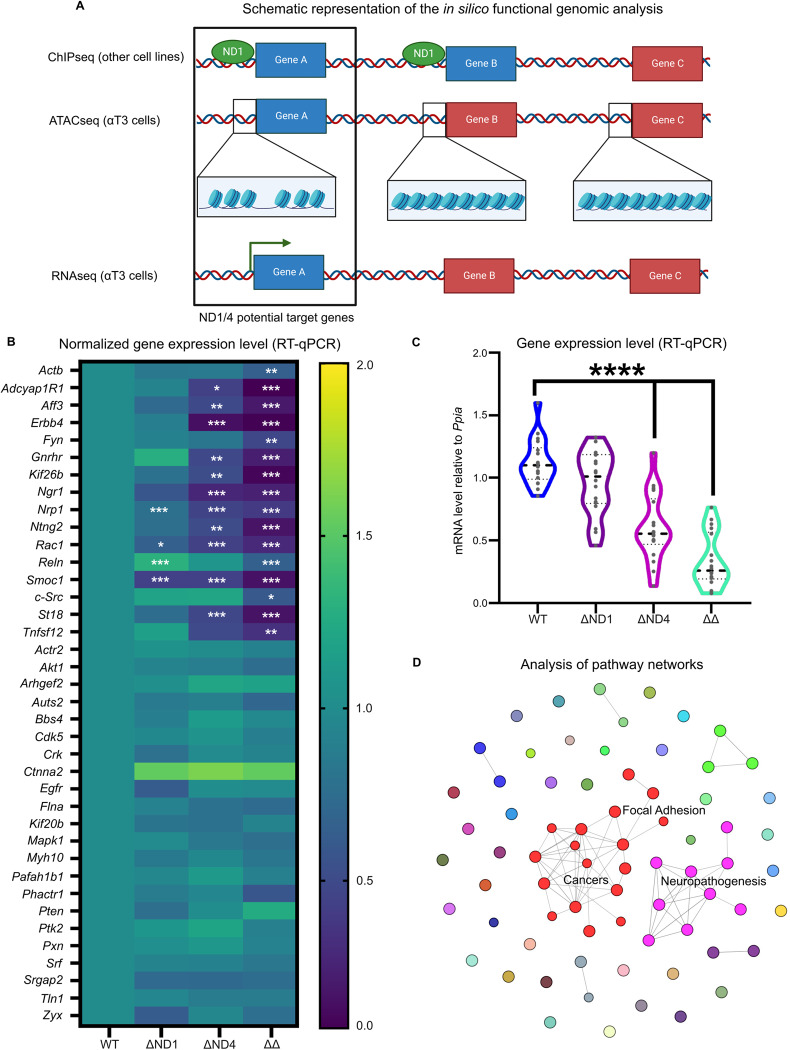
Characterization of the ND1/4 potential regulome in αT3-1 cells. **A** Schematic representation (created with BioRender) of the in silico functional genomics analysis used to identify ND1/4 potential target genes in αT3-1 cells. Expressed genes associated with genomic regions displaying chromatin accessibly and potentially directly targeted by NEUROD factors were identified by cross-referencing ND1 ChIP-seq (from heterologous cell types) with αT3-1 cell ATAC-seq and RNA-seq databases. Retained candidate genes are depicted using blue boxes whereas discarded genes are depicted by red boxed. **B** Heatmap of the expression of 38 ND1/4 potential target genes identified in silico, in WT, ΔND1, ΔND4, and ΔΔ cells. Gene expression levels were normalized to *Ppia*. Color scale represents the normalized expression mean of four independent experiments. Gene expression in ΔND1, ΔND4, ΔΔ cells were compared to WT (values set to 1) by ANOVA followed by Dunnett’s multiple comparison test. *p < 0.05, **p < 0.01, and ***p < 0.001. **C** Violin plots of relative mRNA levels of 16 identified ND1/4 bona fide target genes (genes with significant expression decrease) according to WT, ΔND1, ΔND4, or ΔΔ genotype. To assess the global effect of *Neurod1* and *Neurod4* deletion on gene expression, mean expression levels of the target genes in ΔND1, ΔND4, and ΔΔ genotypes were compared to WT using an ANOVA followed by Dunnett’s multiple comparison test. ****p < 0.0001. **D** Network pathway analysis representation using Cytoscape ClueGO software. Analysis was performed on potential ND1/4 target genes identified by the genomic in silico analysis. Each colored dot is a different pathway regrouping several potential ND1/4 target genes. Related pathways (sharing more than 25% of their genes) are connected by a gray line. Pathways in the same cluster share a common color. Pathway’s name and associated genes are described in supplementary table 1.

In order to characterize the molecular function of ND1 and ND4 in αT3-1 cells, we conducted an in silico functional genomics analysis (Fig. 2A). By cross-referencing several databases, we identified αT3-1 cells expressed genes (RNA-seq data [20]) associated with potential *cis-*regulatory regions displaying chromatin accessibility (αT3-1 cells ATAC-seq [21]) also known to be ND1 targets in other cell types (ND1 ChIP-seq data retrieved from https://chip-atlas.org/ [22]).

In order to validate ND1/4 potential target genes identified in αT3-1 cells by this in silico approach, we measured the expression of a sample of them by RT-qPCR in WT, ΔND1, ΔND4, and ΔΔ cells (Fig. 2B). The expression of 40% of these genes was significantly decreased in ΔΔ cells as compared to WT, indicating that the in silico approach successfully captured a significant proportion of ND1/4 targets in αT3-1 cells. To compare the mean effect of ND1 and ND4 deletion on regulation of gene expression, we plotted the extent of variation according to the cell genotypes (Fig. 2C). This revealed that ND4 might play a more critical role as a transcriptional regulator as compared to ND1 in gonadotrope cells, even if ND1 may still be able to compensate in part for ND4 insufficiency (ΔΔ vs ΔND4 cells, *p* < 0.01).

To identify ND1/4 functions in gonadotrope cells, we then performed a network pathway enrichment analysis on their potential target genes, using Cytoscape ClueGO software [23]. We identified 72 significantly enriched pathways (Fig. 2D and supplementary table 1) organized in two major networks: one clustering neuro-pathogenesis-related pathways, consistently with known ND1/4 functions, and a second network of cancer and focal adhesion-related pathways.

### NEUROD1/4 regulate gonadotrope cell adhesion and motility

**Fig. 3:**
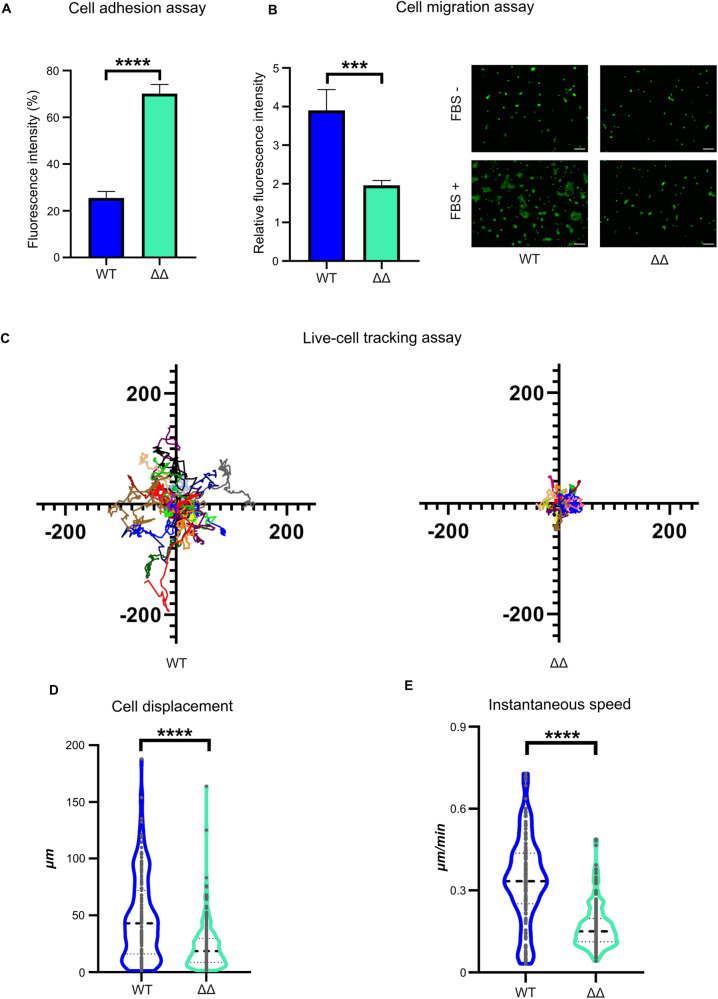
Regulation of αT3-1 cell adhesion and motility by ND1/4. **A** Cell adhesion assay. Fluorescent Calcein AM-labeled WT and ΔΔ cells were seeded on an uncoated 96-well transparent bottom plate for 30 min. Fluorescence intensity is the ratio of fluorescence after PBS flushing over the initial fluorescence, reflecting the fraction of attached cells. Data are the normalized mean ± SEM of 3 independent experiments. The % of ΔΔ attached cells was compared to the one of WT using a Student’s *t* test. ****p < 0.0001. **B** FluoroBlok transwell migration assay. Calcein AM-labeled WT and ΔΔ cells were seeded in FluoroBlok transwell inserts and treated with foetal bovine serum (FBS, 10% in DMEM). Left: Relative Fluorescence Unit is the ratio of post-migratory fluorescence of FBS treated over untreated cells. Data are the normalized mean ± SEM of 6 independent experiments. Migration of ΔΔ cells was compared to WT using a Student’s *t* test. ***p < 0.001. Right: Representative images of post-migratory fluorescent WT and ΔΔ cells with or without FBS as chemoattractant. scale bar = 100 µm. **C** Live-cell tracking assay. WT and ΔΔ cells were transfected with H2B-mCherry to label and track the nuclei. Cells were seeded on Matrigel-coated culture surface until attachment and cell motility was tracked using live video time-lapse microscopy over 16 h. Tracks were analyzed using ImageJ TrackMate. Each graph corresponds to 30 representative tracks of transfected WT or ΔΔ cells with the initial position of the cells centered at the origin. Units: µm. **D** Total cell displacement analysis. The total distance (in µm) traveled by cells over 16 h was analyzed using ImageJ TrackMate on 150 WT and 220 ΔΔ cells. ΔΔ cell mean displacement was compared to WT. ****p < 0.0001 (Mann–Whitney test). **E** Cell velocity analysis. Cell instantaneous speed (nm/s) was calculated from the analysis of 150 WT and 220 ΔΔ cells using ImageJ TrackMate. ΔΔ cell mean velocity was compared to WT. ****p < 0.0001 (Mann–Whitney test).

The in silico analysis suggests that ND1/4 could be implicated in the regulation of focal adhesion (FA) organization and thus in adhesion and motility of gonadotrope cells. In order to test such hypothesis, we first performed a cell attachment assay using WT and ΔΔ cells. We found a 3-fold increase in ΔΔ cell attachment as compared to WT cells (Fig. 3A), suggesting that ND1/4 contribute to the regulation of cell interaction with the culture surface. Given that cell-matrix adhesion is involved in cell motility, we then performed a transwell migration assay using fetal bovine serum (FBS) as chemo-attractant. We observed that ∆∆ cells displayed a significant 2-fold migration decrease (Fig. 3B) whereas migration was unaltered in ΔND1 and ΔND4 cells (supplementary Fig. S1).

In order to further characterize cell motility, we analyzed WT and ΔΔ cell velocity and overall cell displacement using video time-lapse microscopy. While αT3-1 WT cells were motile, ΔΔ cells only wobbled at their initial position (Fig. 3C and supplementary movies M1 and M2). We observed a significant ~50% decrease of both parameters in ΔΔ cells (Fig. 3D, E), indicative of cell motility impairment.

Altogether, these results demonstrate, for the first time, that ND1/4 are critical regulators of motility and adhesion of immature gonadotropes cells.

### NEUROD1/4 regulate focal adhesion number

**Fig. 4:**
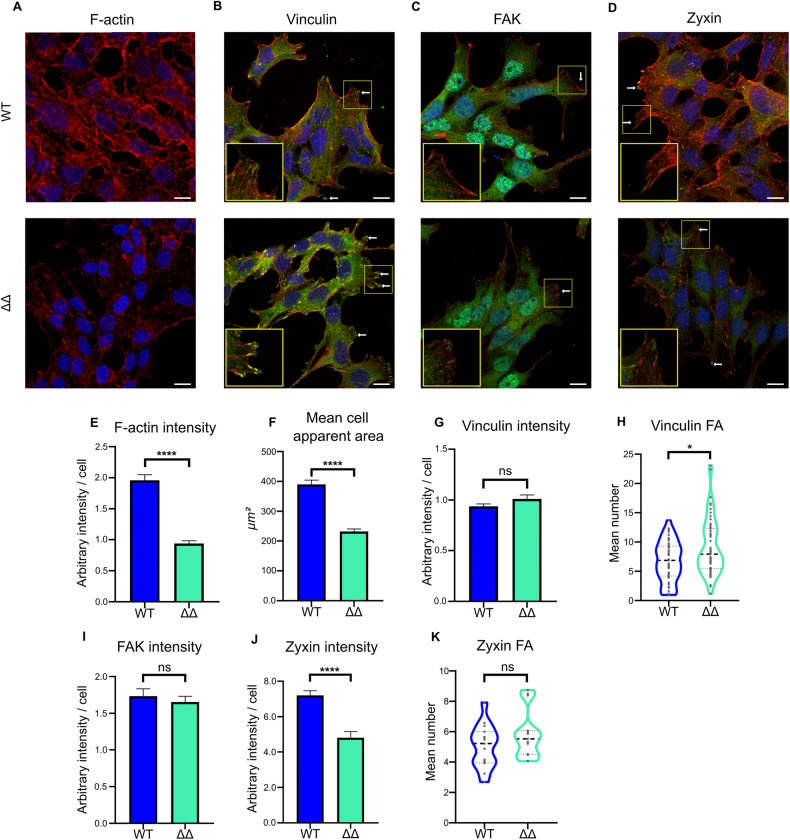
Subcellular localization of actin, vinculin, FAK, and zyxin in WT and ΔΔ cells. **A**–**D** Representative confocal images of **A** F-actin, **B** VIN, **C** FAK, and **D** ZYX-labeled WT and ΔΔ cells. Fixed cells were labeled using phalloidin-TRITC for F-actin and DAPI for nuclei (Red: F-actin, Blue: nuclei, Green: VIN, FAK, or ZYX). **B**, **D** White arrows indicate positive **B** VIN and **D** ZYX-labeled FAs. Areas boxed in yellow are presented in the insets. scale bar = 10 µm. **E** Bar graphs of the mean F-actin fluorescence per cell and **F** mean cell apparent area, quantified on 30 different images for each genotype. ****p < 0.0001 (Mann–Whitney test). **G** Bar graphs of the mean VIN fluorescence intensity per cell and **H** violin plots of VIN-labeled FA particle number, quantified on 50 different images for each genotype. Mean VIN intensity and number of VIN-positive FAs were compared between ΔΔ and WT cells. *p < 0.05 and ns: not significant (Mann–Whitney test). **I** Bar graph of the mean FAK signal fluorescence intensity per cell, quantified on 15 different images for each genotype. Mean cell FAK intensity was compared between ΔΔ and WT cells. ns: not significant (Mann–Whitney test). **J** Bar graph of the mean ZYX signal fluorescence intensity per cell and **K** violin plots of ZYX-labeled FA particle numbers were quantified on 15 different images for each genotype. ZYX- mean cell intensity and number of ZYX-positive FAs were compared between ΔΔ and WT. ****p < 0.0001 and ns: not significant (Mann–Whitney test).

To test the potential regulation of FAs by NEUROD1/4, we first immuno-stained key components of FAs such as vinculin (VIN), FA kinase (FAK) or Zyxin (ZYX) in WT and ΔΔ cells. F-actin cytoskeleton was labeled using Phalloidin-TRITC. F-actin, FAK, and VIN can be considered as constitutive elements of all types of FAs, whereas ZYX is described as a marker of mature FAs [24]. We observed a 50% decrease of F-actin cellular content in ΔΔ cells (Fig. 4A, E), consistent with the decrease in *Actb* expression (Fig. 2A). Yet, actin-based cytoskeleton structures were still observable overall. This analysis also revealed a significant 40% reduction of mean apparent cell area in ΔΔ cells (Fig. 4F), suggesting a reduction of cell spreading. To analyze FAs more specifically, we first immuno-localized VIN and observed a significant 35% increase in number of Vinculin patches in ΔΔ cells without variation of global signal intensity (Fig. 4B, G, H). This suggests that FA number is increased in ΔΔ cells, in line with cells being more adherent and also less motile. To rule out the involvement of other structures implicated in cell adhesion and motility such as lamellipodia and invadopodia, we also analyzed Cortactin distribution which showed no difference between WT and ΔΔ cells (supplementary Fig. S2). We then analyzed the distribution pattern of total FAK and observed a comparable nucleo-cytoplasmic staining with signal at FAs in WT and ΔΔ cells (Fig. 4C, I). This suggests that FAK is similarly expressed in both cell lines. This was further confirmed by RT-PCR and western blotting (supplementary Fig. S3A, B). Finally, we analyzed the distribution pattern of the mature FA marker ZYX (Fig. 4D, J, K). We found an unchanged number of ZYX-labeled FA in ΔΔ cells despite a significant 35% decrease in ZYX global cell content, suggesting that ΔΔ cells might accumulate immature FAs.

Altogether, these results thus indicate that ND1/4 regulate actin cytoskeleton organization and FA number in gonadotrope cells and might be implicated in FA maturation.

### NEUROD1/4 regulate focal adhesion signaling

FA maturation is known to be regulated by phosphorylation of several effectors, such as FAK and c-Src. Briefly, upon stimulation, FAK dimerizes and auto-phosphorylates on Y^397^ residue (as reviewed in [25]). Activated c-Src is then recruited at FAs and, acting as a scaffold, independently of any catalytic activity, facilitates Y^861^ phosphorylation and full FAK activation by Y^576/577^ phosphorylation [26]. c-Src catalytic activity is then required for FAK Y^925^ phosphorylation [26], promoting FA turn-over [27].

**Fig. 5:**
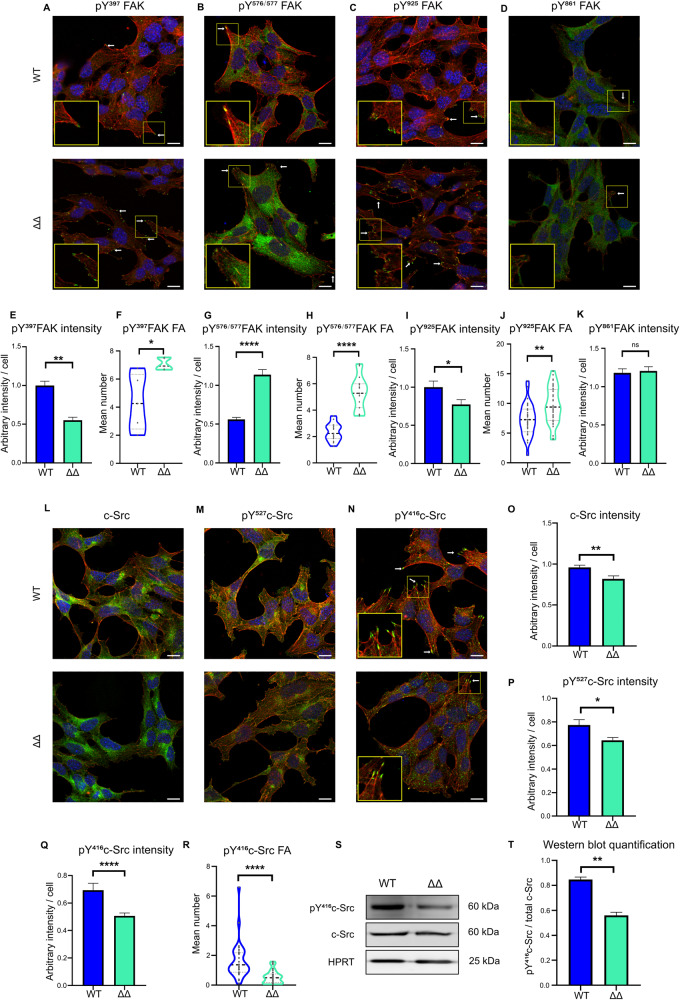
Subcellular localization of phospho FAK, c-Src, and phospho c-Src in WT and ΔΔ cells. **A**–**D** Representative confocal images of pFAK-labeled WT and ΔΔ cells. Phospho FAKs were immuno-localized using **A** pY^397^, **B** pY^576/577^, **C** pY^925^, and **D** pY^861^ antibodies on fixed cells, labeled with phalloidine-TRITC and DAPI (Green: pFAK, Red: F-actin, Blue: Nuclei). White arrows indicate positive FAs. Areas boxed in yellow are presented in the insets. scale bar = 10 µm. **E**–**K** Fluorescent signal quantification: **E**, **G**, **I**, **K** Bar graph of the mean pFAK fluorescence intensity per cell. **F**, **H**, **J** Violin plots of the number of pFAK-positive FAs. Quantification was performed on 5 (pY^397^), 15 (pY^576/577^), 35 (pY^925^), and 15 (pY^861^) different images for each genotype. For pY^861^ FAK, displaying cytoplasmic signal but only a weak signal at FAs, only mean cell fluorescence quantification was conducted. Mean cell fluorescence intensity and/or positive FA number were compared between ΔΔ and WT cells. *p < 0.05, **p < 0.01, ****p < 0.0001 and ns: not significant (Mann–Whitney test). **L**–**N** Representative confocal images of c-Src, pY^527^, and pY^416^c-Src-labeled WT and ΔΔ cells. c-Src and phospho c-Src were immuno-localized using **L** total c-Src, **M** pY^527^, **N** pY^416^ antibodies on fixed cells, labeled with phalloidine-TRITC and DAPI (Green: c-Src or phospho c-Src, Red: F-actin, Blue: Nuclei). White arrows indicate positive FAs. scale bar = 10 µm. **O**–**R** Fluorescent signals quantification: for total c-Src and pY^527^c-Src, displaying cytoplasmic signal but only a weak signal at FAs, only mean cell fluorescence conducted. For pY^416^c-Src, displaying both cytoplasmic signal and intense signal at FAs, mean cell fluorescence intensity and particle analysis were conducted. **O**–**Q** Histograms of mean c-Src or pY^527^ or pY^416^ signal fluorescence intensity per cell and **R** violin plots of pY^416^ c-Src-labeled FAs number were quantified on 20 (c-Src), 10 (pY^527^) and 30 (pY^416^) different images of both WT and ΔΔ cells. Mean cell fluorescence intensity and positive FA number were compared between ΔΔ and WT. *p < 0.05, **p < 0.01, and ****p < 0.0001 (Mann–Whitney test). **S** Representative image of western blot analysis of total c-Src and phosphorylated Y^416^c-Src in WT and ΔΔ cells. Intensity ratio: ratio of pY^416^c-Src over total c-Src band intensities. **T** Intensity ratio was compared between ΔΔ and WT using a Student’s *t* test. **p < 0.01.

We analyzed here both FAK phosphorylation level and cellular distribution for all aforementioned phosphorylation marks. We observed a significant increase in the number of FAs positive for pY^397^, pY^576/577^, and pY^925^ FAK in ΔΔ cells (Fig. 5A–D, F, H, J), consistently with the overall increase in FA number in these cells. Concerning FAK phospho-forms cellular content, we observed a complex perturbation pattern in ΔΔ cells: pY^861^ level was similar between ΔΔ and WT cells, while both pY^397^ and pY^925^ were significantly decreased and pY^576/577^ was significantly increased in ΔΔ cells (Fig. 5E, G, I, K). Similar level in FAK pY^861^ rules out a systematic impairment of FAK phosphorylation. Decreased level of pY^397^ might suggest a defect of FAK early activation and might be due to a lesser cell spreading (Fig. 4F) and subsequent integrin activation. However, increased level of pY^576/577^ might indicate a secondary hyper-activation of FAK and suggests that c-Src scaffolding activity is intact in these cells. Finally, decrease in pY^925^ level is consistent with a defect in FA late maturation and suggests an impairment in c-Src kinase activity. Altogether these results suggest that FA maturation defect might disturb FAK phosphorylation dynamics. To better understand this complex perturbation pattern, we then investigated c-Src activation.

At the basal state, c-Src is phosphorylated on Y^527^, a repressive post translational modification. Dephosphorylation of pY^527^ is necessary for c-Src auto-phosphorylation on Y^416^ which leads to its activation [28]. We thus analyzed total and phosphorylated c-Src localization in WT and ΔΔ cells (Fig. 5L–R). Total and pY^527^c-Src displayed a cytoplasmic localization whereas pY^416^c-Src localized at FAs. We observed a significant, albeit slight, decrease in total or pY^527^c-Src contents between WT and ΔΔ cells, whereas there was an unexpected 50% decrease of pY^416^ labeled-FAs in ΔΔ cells. Hypo-phosphorylation of c-Src on Y^416^ was confirmed by western blot (Fig. 5S, T). The c-Src activation defect is consistent with the FAK phosphorylation pattern described above, suggesting that ND1/4 are implicated in c-Src activation and hence in FA organization.

### NEUROD1/4 regulate gonadotrope cell motility through c-Src activation

**Fig. 6:**
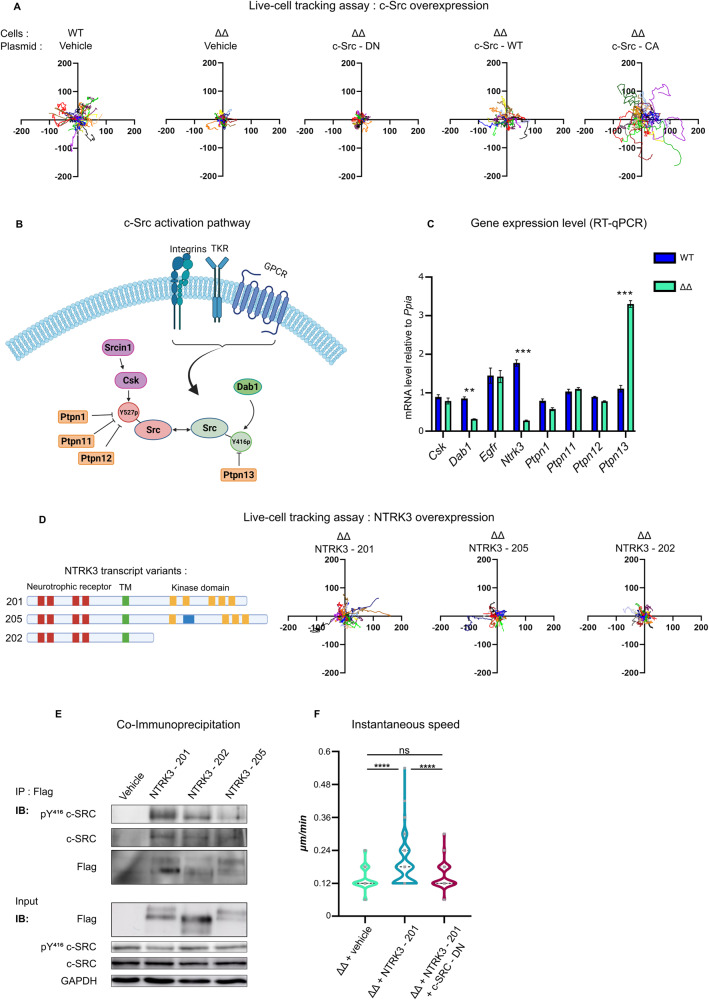
ΔΔ cells motility defect is rescued by an NTRK3-c-Src signaling pathway. **A** Cell motility rescue assay by c-SRC. WT and ΔΔ cells were transfected with H2B-mCherry to label and track the nuclei. WT cells were co-transfected with GFP expression plasmid (vehicle) and ΔΔ cells were co-transfected with either GFP (vehicle), c-Src DN, c-Src WT, or c-Src CA expression plasmid. Each graph corresponds to 30 representative tracks of transfected WT or ΔΔ cells with the initial position of the cells centered at the origin. **B** Schematic representation (created with BioRender) of c-Src inhibition and activation mechanisms. c-Src inhibition involves both Y^527^ phosphorylation by the CSK kinase and Y^416^dephosphorylation by the PTPN13 phosphatase. Upon extracellular signaling by integrins, c-Src disengages from CSK, with concomitant Y^527^dephosphorylation that may involve several putative phosphatases, and finally c-Src autophosphorylates on Y^416^. Signal transducing adaptor proteins such as DAB1 (Disabled-1) are known to increase c-Src activation. Finally, membrane receptors such as the neurotrophin 3 tyrosine kinase receptor NTRK3 have also been shown to trigger c-Src activation. **C** Gene expression of the actors of c-Src inhibition and activation pathways in WT and ΔΔ cells. Gene expression levels were normalized to *Ppia*. Data are the normalized mean ± SEM of three independent experiments. Gene expression were compared between ΔΔ and WT cells using a Student’s *t* test. ns: not significant, ***p < 0.001. **D** Cell motility rescue assay by NTRK3. (Left) Schematic representation of the *Ntrk3* isoforms expressed in αT3-1 cells. Ligand domain is depicted with red boxes, the transmembrane domain with a green box and the tyrosine kinase domain with yellow boxes. The modified exon in *Ntrk3-*205 isoform is represented by a blue box. (Right) Cell motility assay. ΔΔ cells were co-transfected with H2B-mCherry and either with *Ntrk3*-201, *Ntrk3*-202, or *Ntrk3*-205 isoform expression plasmids. Each graph corresponds to 30 representative tracks of transfected ΔΔ cells with the initial position of the cells centered at the origin. **E** c-SRC-NTRK3 co-immunoprecipitation assay. WT cells were co-transfected with c-Src-eGFP and either with 3XFLAG control plasmid (lane 1, vehicle) or 3XFLAG-tagged isoform expression plasmids (Lane 2, *Ntrk3*-201, lane 3 *Ntrk3*-202 and lane 4 *Ntrk3*-205). NTRK3 was immunoprecipitated using anti-FLAG M2 antibody-coated magnetic beads. Immuno-precipitates were analyzed by Western blot for NTRK3 precipitation (using anti-FLAG antibody) and for both total and pY^416^ c-Src co-precipitations (Top panels). Expression of FLAG-tagged NTRK3, total c-SRC and pY^416^c-SRC-eGFP were verified by western blot analysis on whole cell lysates (Bottom panels). GAPDH was used as an internal reference for sample loading. The different bands observed for NTRK3 isoforms are consistent with glycosylated forms of the receptor. **F** Cell velocity analysis. ΔΔ cells were co-transfected with H2B-mCherry and either with GFP (vehicle), *Ntrk3-201*, or a mix of *Ntrk3-201* and DN *c-Src* expression plasmids. Cell instantaneous speed (µm/minute) was calculated from the analysis of over 100 transfected ΔΔ cells over 16 h videomicroscopy, using ImageJ TrackMate. ΔΔ cell mean velocity was compared between the three conditions by ANOVA followed by Tukey multiple comparison test. ns: not significant, ***p < 0.001.

To link c-Src hypo-activation to motility defect of ΔΔ cells, we first over-expressed WT, dominant negative (DN) K297R/Y527F or constitutively activated (CA) Y527F c-Src in ΔΔ cells and monitored cell motility using video time-lapse microscopy (Figs. 6A and S4A, B). While DN c-Src had no effect, we observed that over-expression of WT c-Src fully rescued cell motility in ΔΔ cells. Furthermore, CA c-Src transfected ΔΔ cells displayed even greater motility than WT c-Src transfected ones. This suggests that c-Src activation defect is a major component of ΔΔ cells motility impairment.

Activation of c-Src is dependent on a balance between inhibitory and activating pathways regulating the phosphorylation ratio on Y^416^ and Y^527^ residues (Fig. 6B). Both sides involve several actors, such as kinases and phosphatases [28] as well as adaptor proteins such as DAB1 (Disabled-1) [29]. If integrin signaling is the canonical activation pathway [30], several membrane receptors such as the neurotrophin 3 tyrosine kinase receptor NTRK3, have also been described to activate c-Src [31] (Fig. 6B).

We investigated the pathways regulating c-Src activation by RT-qPCR in ΔΔ cells. Interestingly, we observed a strong 7-fold decrease in *Ntrk3* expression associated with a decrease in *Dab1* and an increase in *Ptpn13* transcript levels (Fig. 6C). All of these changes potentially contribute to the decrease in c-Src activation in ΔΔ cells.

We then evaluated the relative importance of DAB1, PTPN13, and NTRK3 in ΔΔ cell motility defects. Over-expression of DAB1, either its WT form or a phospho-mimetic activated Y185/197D mutant [32], failed to significantly rescue ΔΔ cell motility. The same was observed with *Ptpn13* knock-down by CRISPRi (Figs S4C–E), ruling out a major contribution of DAB1 or PTPN13 in ΔΔ cell motility defect. We then over-expressed NTRK3 in ΔΔ cells and assessed motility. According to RNA-seq data, αT3-1 cells express three NTRK3 isoforms (Fig. 6D): *Ntrk3-201* (Ensembl browser transcript name), the full-length active receptor with both neurotrophin binding and tyrosine kinase (TK) domains, *Ntrk3-205*, an isoform with an additional exon in the TK domain leading to functional deficiency [33] and, finally, the inactive *Ntrk3-202* short variant, without the TK domain. Only expression of the full-length *Ntrk3-201* rescued cell motility in ΔΔ cells (Figs. 6D and S4A, B). Interestingly, NTRK3-201 interacts with pY^416^c-Src in αT3-1 cells, as demonstrated by co-immunoprecipitation experiments (Fig. 6E), whereas interactions are noticeably weaker with NTRK3-202 and NTRK3-205 isoforms (2.5-fold reduction compared to NTRK3-201 after normalized quantification). These results suggest that NTRK3 full signaling activity is thus required for c-Src activation. To test whether NTRK3-driven cell motility is dependent on c-Src activity, we measured motility of ΔΔ cells expressing NTRK3 alone or together with DN c-Src. We observed that DN c-Src blocks the positive effect of NTRK3 on cell speed (Fig. 6F), indicating that c-Src mediates NTRK3 effect on cell motility.

Altogether, these results demonstrate that immature gonadotrope cell motility is regulated by a Neurod1/4-Ntrk3-Src activation pathway.

### NTRK3 regulate gonadotrope cell number and position during mouse pituitary development

**Fig. 7:**
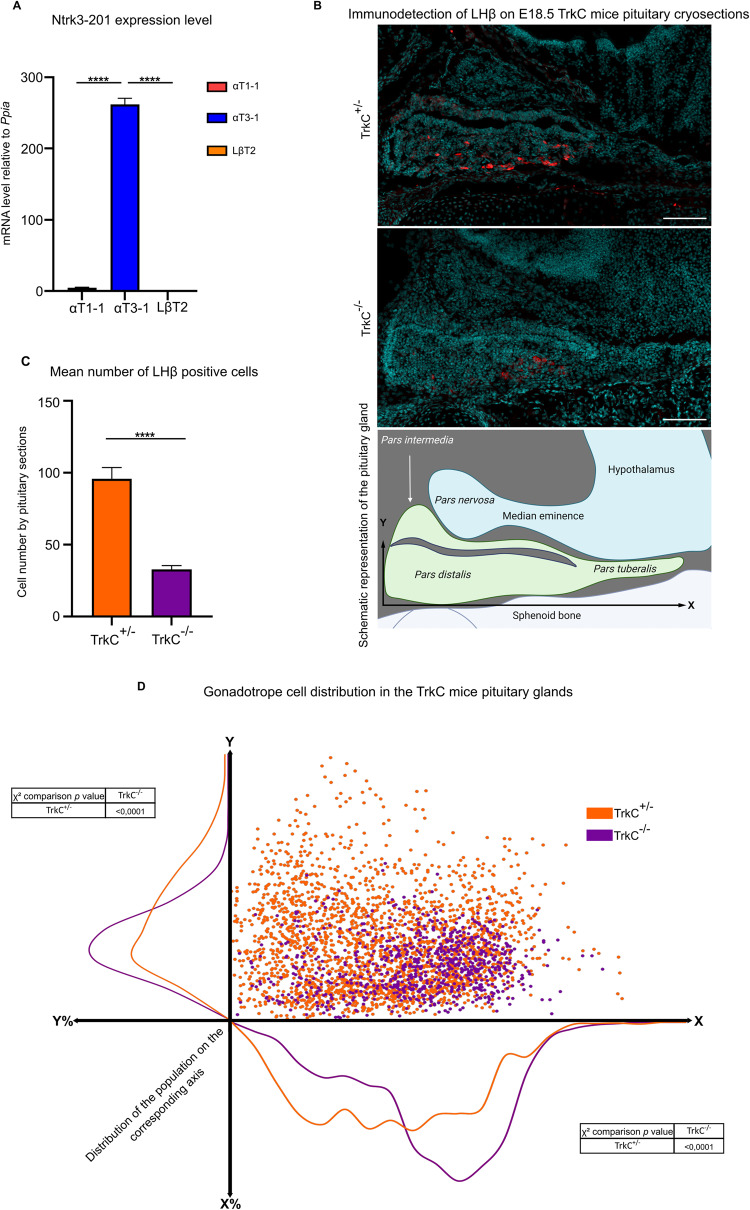
NTRK3 regulates gonadotrope cell positioning in the developing pituitary in vivo. **A** *Ntrk3* active isoform expression in the in vitro model of gonadotrope differentiation. *Ntrk3-201* expression level was quantified by qPCR and normalized to *Ppia*. Data are the normalized mean ± SEM of three independent experiments. *Ntrk3-201* expression level was compared to αT3-1 cells using an ANOVA followed by Dunnett’s multiple comparison test. ****p < 0.0001. **B** Gonadotrope cell population distribution in TrkC mouse embryonic pituitary at E18.5. LH beta sub-unit (LHβ), specifically expressed by gonadotrope cells, was detected by immunohistofluorescence on E18.5 TrkC^+/−^ and TrkC^−/−^ embryo cryosections. Nuclei were stained by DAPI. Top and middle panels: Representative images of TrkC^+/−^ (taken as control) and TrkC^−/−^ pituitary sections. scale bar = 100 µm. Bottom panel: schematic representation of the pituitary and surrounding structures at E18.5. The pituitary gland sits between the palate sphenoid bone (ventral) and the *pars nervosa* and median eminence (dorsal). Gonadotrope cells, labeled in red, are mostly localized in the *pars distalis*, the ventral part of the endocrine pituitary. X and Y black arrows: cartesian coordinate system used to analyze gonadotrope cell distributions. **C** Quantification of gonadotrope cell number in TrkC^+/−^ and TrkC^−/−^ embryonic pituitary at E18.5. LHβ-expressing gonadotrope cells were numerated on a minimum of six sections of three female individuals for each genotype. Histological sections were matched between genotypes for similar anatomical positions. Only sections with complete pituitary of the same surface, and containing both *pars nervosa* and median eminence were retained to minimize counting bias. Number of gonadotrope cells per sections were compared between genotypes. ****p < 0.0001 (Mann–Whitney test). **D** Distribution of the gonadotrope cell population within the pituitary of TrkC^+/−^ and TrkC^−/−^ embryos at E18.5 (Green; TrkC^+/−^ and red: TrkC^−/−^). Each identified gonadotrope cell on each section was plotted on a standardized cartesian coordinate (X;Y) system with the antero-posterior embryo axis represented by “X” and the dorso-ventral axis by “Y”. For each genotype, histograms of gonadotrope cell distributions on both X and Y axis were estimated. Distribution on each axis were compared using *χ*^2^ comparison test.

To investigate a potential role of *Ntrk3* during gonadotrope differentiation, we first analyzed *Ntrk3-201* transcript levels in the in vitro model of gonadotrope differentiation by RT-qPCR (Fig. 7A). We observed that the active isoform of *Ntrk3* is specifically expressed in αT3-1 cells, representative of E14 to E16 gonadotrope cells.

Gonadotrope cells appear around E13.5 in the mouse developing pituitary, in a restricted ventral area of the *pars distalis* [34]. This population then expands in the pituitary gland and, at E18.5, gonadotrope cells are organized in structured networks throughout the whole anterior lobe of the pituitary [35]. This expansion during pituitary organogenesis might implicate both proliferation and migration events. To investigate a potential role of *Ntrk3* in gonadotrope cell expansion, we took profit of a *Ntrk3* constitutive knock-out mouse model (TrkC^tm1a^, [36]), and analyzed the gonadotrope cell population in E18.5 TrkC^+/−^ (taken as control) and TrkC^−/−^ embryos by immunostaining of LHβ, a specific marker of gonadotrope cells. We first observed a significant 3-fold decrease in the number of LHβ positive cell in TrkC^−/−^ embryos as compared to heterozygous controls (Fig. 7B, C), suggesting that NTRK3 might be implicated in pituitary colonization. To test for a regulatory role of NTRK3 on cell motility, we then analyzed the distribution of gonadotrope cells in the pituitary along the antero-posterior and on the dorso-ventral axes. We observed that gonadotrope cells spread significantly less on both axes in TrkC^−/−^ fetal pituitaries as compared to TrkC^+/−^ (Fig. 7B, D), suggesting that NTRK3 might be implicated in gonadotrope cell migration in vivo.

These results suggest that NTRK3 is an important actor of gonadotrope population organization in the developing pituitary, being implicated in population size as well as in proper colonization of the pituitary anlage.

## Discussion

The concept of a tridimensional organization of endocrine cell networks in the pituitary gland has only recently emerged [16] and gonadotrope cell networks are now believed to be crucial for proper regulation of reproduction. However, the molecular mechanisms regulating gonadotrope cell motility and, *in fine* the structuration of the endocrine population, remain mostly elusive.

**Fig. 8:**
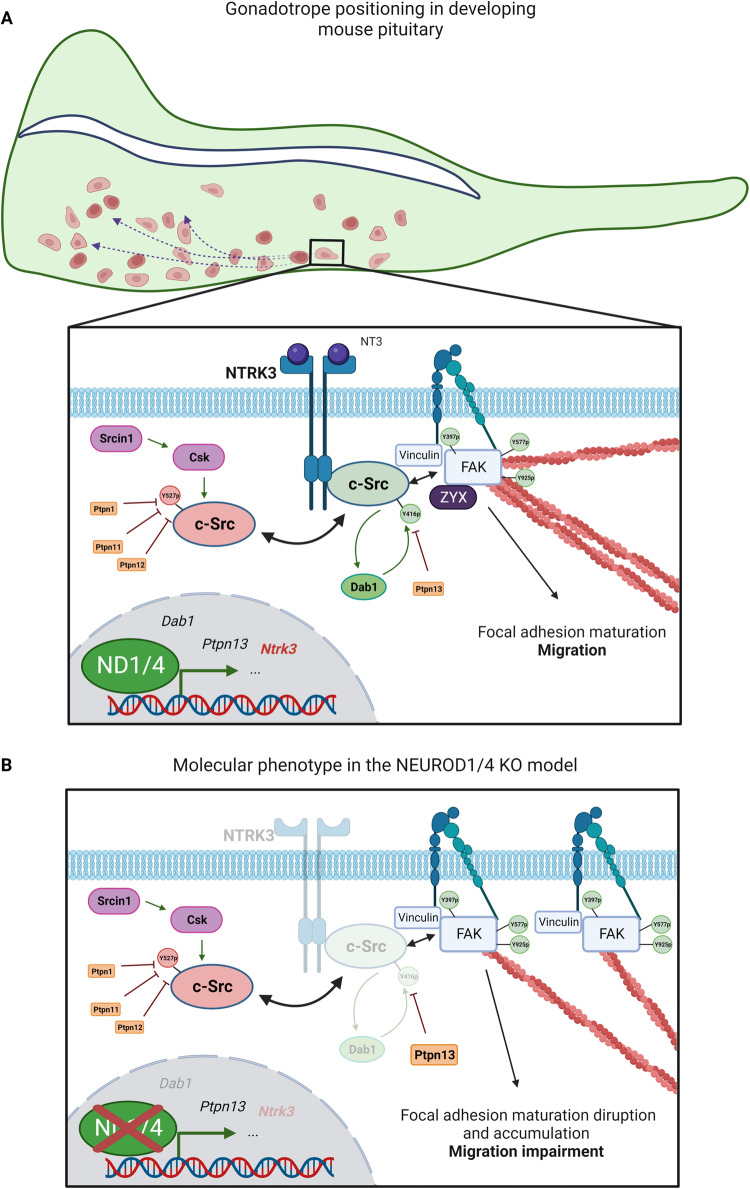
The Neurod1/4-Ntrk3-Src pathway regulates gonadotrope cell motility during gonadotrope differentiation. Schematic representation of the principal results of this study (created with BioRender). **A** Gonadotrope cells (in red) differentiate in the ventral part of the pituitary and then disseminate in the gland. Our work shows that gonadotrope cell motility during pituitary organogenesis is dependent on NTRK3 receptor whose expression is controlled by ND1 and ND4 transcription factors. NTRK3 interacts with c-Src kinase and promotes its activation, a prerequisite for maturation of FAs and cell migration. **B** NEUROD1/4 insufficiency leads to a defect in NTRK3 expression, leading to a c-Src hypo-activation, which, in turn, disturbs FAK phosphorylation pattern. These signaling disruptions induce an accumulation of immature focal adhesions, preventing cell migration.

In the current work, we investigated the molecular regulation of gonadotrope cell motility. Using an in silico functional genomic analysis combined with CRISPR/Cas9 cellular knock-out, we demonstrated for the first time that, during pituitary ontogenesis, immature gonadotrope cell motility is regulated by a molecular pathway involving the ND1/4 transcription factors, the neurotrophin NTRK3 receptor and c-Src kinase (Fig. 8A).

In gonadotrope cells, ND1/4 stimulate NTRK3 expression, which in turn interacts with and activates c-Src. Full c-Src kinase activity would be then required for optimal FAK phosphorylation, leading to FA terminal maturation and disassembly. ND1/4 insufficiency, leading to a hypo-activation of c-Src kinase activity, while not affecting its scaffolding role at FAs, unbalances both FAK phosphorylation pattern and FAK distribution, leading to immature FAs accumulation and cell motility defects (Fig. 8B).

Mutation of ND1 and ND4 in gonadotrope cells affects expression of other genes of the c-Src activation pathway such as DAB1 or PTPN13. However, we showed here that, amongst them, only NTRK3 efficiently rescued cell motility, suggesting that, in immature gonadotrope cells, NTRK3 activation of c-Src might be essential to trigger cell motility.

Taking advantage of a knock-out mouse model, we further demonstrated that NTRK3 receptor is crucial to ensure the colonization of the pituitary gland by gonadotrope cells during embryogenesis. NTRK3 deletion also led to a reduced number of gonadotrope cells that might be explained by a defect in migration, proliferation, or differentiation, in agreement with NTRK3 known functions [37–40].

Because ND1, ND4, and NTRK3 are expressed in other cell types, the Neurod1/4-Ntrk3-Src pathway might also be mobilized in cells requiring motility processes throughout their differentiation. For example, this pathway is expressed in human testicular spermatids according to the human protein atlas database and, although spermatid motility is an important part of their differentiation [41], the underlying molecular mechanisms are still poorly understood.

Whereas both ND1 and NTRK3 have already been reported to regulate cell motility [39, 42–45], much less was known about ND4 cellular function. We showed here that ND4 is as potent as ND1 to regulate gonadotrope cell motility. The transcriptional activity of ND4 would, however, be more important than ND1 for gonadotrope cells since ND1 only partially compensates for ND4 absence.

Pituitary gonadotrope cells display a remarkable functional plasticity during adulthood, which is essential to regulate fertility. Gonadotrope cell networks are suspected to play a important role in this plasticity as they are reconfigurated under physiological challenges such as puberty and lactation [46]. However, little is known about the regulation of gonadotrope cell motility [47, 48] or migratory ability [49] during these events. Interestingly NTRK3 remains expressed in adult gonadotrope cells, as demonstrated by single-cell transcriptomic analysis of adult rat pituitaries [50]. NTRK3, which has recently been implicated in neural network formation [51], could thus be a major actor of gonadotrope cell network plasticity during reproductive life. It would be thus interesting to analyze NTRK3 signaling in adult pituitary during critical physiological demands.

Dysregulations of NTRK3 signaling are known to be implicated in numerous cancers [52]. Gonadotrope adenomas are frequent cancers accounting for approximately 40% of all pituitary adenomas [53, 54]. However, most gonadotrope adenomas are poorly characterized and the molecular etiology of gonadotrope cell carcinogenesis is mostly unknown. A screen of NTRK3 mutations in these cancers should open new therapeutic avenues.

In conclusion, our work provides new insights into the molecular pathways controlling gonadotrope cell positioning during their differentiation in the developing pituitary and should contribute to a better understanding of the role of gonadotrope cell networks. Further studies are needed to establish whether this pathway is also mobilized at adulthood and contributes to the adaptation of gonadotrope cell population to physiological challenges at the critical stages of the reproductive life.

## Materials and methods

### Materials

Primary and secondary antibodies along with primers used for RT-qPCR, PCR genotyping, *cis-*regulatory regions, gRNAs, and ORFs cloning are described in supplementary table 2.

### Cell cultures

The αT1-1, αT3-1, and LβT2 mouse gonadotrope cell lines (generously given by P. Mellon, University of California, La Jolla, CA) were grown in high-glucose DMEM supplemented with 10% fetal bovine serum (FBS from Gibco) and 0.1% penicillin/streptomycin, at 37 °C with 5% CO_2_. Cells are regularly tested for mycoplasma and for proper cell identity.

### In silico analysis

NEURDO1 ChIP-seq Bed files (GSM3579940 [8] and GSM1306336 [55]) were retrieved from the ChIP-atlas website [22] and cross-referenced for overlapping target regions with the αT3-1 ATAC-seq [20] using bedtools intersect [56]. Only NEURDO1 ChIP-seq data were used as there is no available ChIP-seq data for NEURDO4 in the mouse. Genomic regions were then associated with the nearest gene Transcription Start Site using Homer annotatePeaks.pl [57]. Pathway analysis and network clustering were performed using Cytoscape ClueGO software [23].

### RNA extraction and mRNA quantification

Total cellular RNAs were isolated using *TRIzol*™ Reagent according to the manufacturer’s protocol. RNAs (2 μg) were reverse transcribed with SuperScript II reverse transcriptase (ThermoFisher Scientific) using random primers according to the manufacturer’s instructions. qPCR quantifications were performed using Takyon™ 2X MasterMix from Eurogentec. Experiments were conducted independently in at least 3 replicates.

### Immunocytofluorescence

Cells were seeded at a density of 500 cells/mm² on Nunc® *Lab*-*Tek*®. Coating was performed using Corning® Matrigel® Growth Factor Reduced (GFR) basement membrane matrix diluted in PBS (1/50) for 30 min at 37 °C. Cells were fixed 48 h after plating, with 4% PFA for 15 min, and 0.1% Triton X-100 permeabilization was performed. Permeabilized cells were then incubated with primary antibodies overnight at 4 °C and then with Alexa488 secondary antibodies 1 h at room temperature (RT). F-actin was labeled using Rhodamin-Phalloidin (ThermoFisher Scientific) and nuclei were labeled using DAPI.

Images were acquired using the ZEISS LSM 700 confocal microscope on the Functional and Adaptive Biology Unit microscopy facility, using laser settings and digital gain as described for each antibody in the additional file 2.

### Image analyses

Images were analyzed using the ImageJ software [58]. For each image, cell number was assessed by counting the number of DAPI positive nuclei.

According to the signal patterns, we either quantified total intensity of fluorescence and/or FA number by particle analysis. Intensity of fluorescence was quantified using the “measure” option of the ImageJ software, after setting a minimum threshold value of 5 for each image. Raw Intensity values obtained were then compared to the number of nucleus per image to assess an approximated value of intensity for each cell type. Mean cell surface was assessed using the “measure” option of the ImageJ software, after setting a minimum threshold value of 5 for each image. Area values obtained were then normalized to the number of nucleus per image to evaluate the approximated surface covered by each cell type. Particle quantification was performed using the ImageJ software by reducing background for both actin and immuno-labeled protein signals with a Rolling ball radius of 2.5 pixels. Then, threshold of the immuno-labeled protein channel was set using the “Auto Local Threshold” tool with the “Phansalkar” method (radius of 25 and specific parameters value of 0.1). Actin and immuno-labeled protein colocalization was then assessed using the “Colocalization Threshold” tool. Colocalizing particles were analyzed with the “Analyze Particles” tool using the following parameters: “size: 0.3 pixel^2^ – infinity” “circularity: 0.0–0.8”. The number of particles counted was then normalized to the number of nuclei within each image.

Particle analysis for FAK pY^397^ and FAK pY^925^, displaying a high signal to background ratio at FA, was performed using the Analyze Particles command directly on the immuno-labeled protein channel without prior actin colocalization. Only objects with size ranging from 0.2 to 5 pixel^2^ and circularity parameter ranging from 0.0 to 0.7 were considered. Finally, the total count of particles for each image was normalized to the nucleus number.

### Luciferase reporter assay

Cloning of the potential *cis-*regulatory regions of *Neurod4:* potential *cis*-regulatory element genomic regions were amplified from mouse DNA. *Neurod4* proximal promoter (R2 region) was cloned upstream the luciferase gene in the pGL4.12 backbone (Promega). R1, R3, R4, R5, and R6 potential enhancers were then cloned in the pR2-GL4 vector downstream the SV40 poly(A) signal, between the BamH1 and SalI sites. Cell transfection was performed as follows: briefly, 50,000 cells were transiently transfected in 96-well plates using Lipofectamine ® 3000 (ThermoFisher Scientific) with 100 ng/well of pGL4 plasmids along with 5 ng/well of pRL-SV Renilla plasmid as an internal control for normalization. After 48 h transfection, firefly and Renilla luciferase activities were measured using the Dual-Luciferase Reporter Assay System (Promega). Each experiment was performed six times independently in triplicates. Data were normalized to Renilla for each condition. ANOVA followed by Dunnett’s multiple comparison test was performed independently for each cell line and condition.

### CRISPR/Cas9 deletions of *Neurod1* and *Neurod4* R6 region

pLV hUbC-Cas9-T2A-GFP (#53190), and pSPgRNA (#47108) plasmids [59] were purchased from Addgene. Specific guide RNAs, on both side of *Neurod1* ORF or *Neurod4* R6 enhancer, were designed using CCTop online tool [60] and cloned in pSPgRNA plasmid.

About 5 × 10^6^ αT3-1 cells were electroporated with the Cas9 expression vector and the gRNA couples expression plasmids at a ratio of 1.5:8.5 µg using a Neon^®^ Transfection System (Invitrogen) according to the manufacturer’s protocol (two pulses at 1500 mV for 15 ms). The empty pSPgRNA was used as control. After 48 h, GFP-positive cells were sorted using the FACS Aria II on the PIC2 facility of the Unit of Functional and Adaptive Biology. Sorted cells were then plated at low density until colonies were formed. ΔND1 and ΔND4 singles colonies were then genotyped for the *Neurod1* locus and for the *Neurod4* R6 region, respectively, using primers described in additional file 2. To generate *Neurod1* and *Neurod4* ΔΔ mutants, the R6 enhancer was deleted in a ΔND1 αT3-1 clone following the same protocol.

### Protein extraction and western blot

Cellular proteins were extracted using a modified RIPA lysis buffer (150 mM NaCl; 50 mMTris pH 8; 1% Triton X-100; 0.1% SDS; 1/1000 DNase, supplemented with 1/100 Phosphatase and protease inhibitor cocktail from ThermoFisher Scientific) and separated on a 10% SDS-PAGE. After transfer, nitrocellulose membranes were incubated overnight at 4 °C with primary antibodies against Src and pY^416^Src diluted in Tris-buffered saline containing 0.01% Tween 20 (TBS-T) supplemented with 5% milk. After extensive washing, blots were incubated with a horseradish peroxidase-conjugated secondary antibody (GE healthcare #NA934V) in TBS-T/5% milk for 60 min at RT and then washed. Immunodetection was performed using an enhanced chemiluminescence detection system (GE Healthcare). Uncropped western blot images are shown in supplementary data.

### ORFs cloning

For rescue experiments, the pIREShyg3 backbone (Clonetech) was modified to express a 2A-GFP cassette. *c-Src*, *Dab1*, and *Ntrk3* isoforms coding sequences were amplified from αT3-1 cDNAs and inserted in frame in the p2A-GFP IRES hyg3 backbone. Constitutively activated (CA) and Dominant Negative (DN) c-Src and phopho-mimetic DAB1 were obtained by fusion PCR mutagenesis.

WT *c-Src* was further subcloned in pEGFP N1 backbone in frame with GFP (pSrc-eGFP). A 3X FLAG tag was inserted in Cter of each *Ntrk3* isoforms (pNtrk3-3XFLAG 201, 202 and 205).

### Motility assay

Cells were transfected by electroporation with pH2B-mCherry to track nucleus position (0.5 × 10^6^ cells, 1 µg plasmid, two pulses at 1500 mV for 15 ms). For *c-Src*, *Ntrk3*, and *Dab1* rescue experiments, cells were co-transfected with *c-Src*, *Ntrk3*, or *Dab1* expression plasmids. For *Ptpn13* knockdown, cells were co-transfected with a pLV hUbC-Zim3KRAB hdCas9-T2A-GFP, expressing a dead Cas9 (Addgene #53191) fused with the repressor KRAB domain of the human ZIM3 gene (as described in [61]), and gRNA targeting *Ptpn13* proximal promoter described in [62] and supplementary table 2). Cells were then seeded in 12-well plates coated with 1/50^e^ Corning® Matrigel® GFR at a density of 25,000 cells/cm². mCherry fluorescent nuclei were then followed using video time-lapse microscopy at 37 °C and 5% CO_2_ during 16 h. Cell displacement analysis and tracking were done on the red channel using the ImageJ software with the “TrackMate” plugin, using the following parameters: LoG detector, threshold of 3.0, the HyperStack Displayer, Simple LAP tracker, 75 microns Gap-closing max distance, 15 microns linking max distance and 5 microns Gap-closing max frame gap. Only cells displaying red nuclei signal, green cytoplasmic signal and directly adhering to the matrix through the whole movie were kept for analysis.

### FluoroBlock cell migration assay

150,000 cells/cm² were seeded in the top part of Corning® FluoroBlok™ Cell Culture Inserts in DMEM serum-deprived medium for 24 h. Medium in the bottom chamber was then replaced by 10% FBS DMEM medium to induce cell migration. After 16 h, cells were labeled with the fluorescent Calcein AM dye (ThermoFisher Scientific) in Hank’s Balanced Salt Solution for 2 h. Bottom Fluorescence intensity, corresponding to post-migratory cells was measured using a FlexStation3 microplate reader at λ 495 nm.

### Cell adhesion assay

WT and ΔΔ cells were labeled with Calcein AM dye in Hank’s Balanced Salt Solution for 30 min. Labeled cells were seeded on an uncoated cell surface of 96-well transparent bottom plate for 30 min. Initial bottom fluorescence was read using a FlexStation3 microplate reader at λ 495 nm and then cells were flushed away by two PBS washes. Residual bottom fluorescence was then read and the relative number of attached cells was estimated by calculating the percentage of remaining fluorescence intensity.

### Co-immunoprecipitation

15 × 10^6^ WT αT3-1 cells were co-transfected with c-SRC-eGFP and NTRK3 isoform expression plasmids (pNtrk3-3XFLAG 201, 202 and 205) by electroporation for 24 h. Cellular proteins were extracted using a modified RIPA lysis buffer (150 mM NaCl; 50 mMTris pH 8; 1% Triton X-100 containing 1/100 Phosphatase and protease inhibitor cocktail). Extracts were incubated overnight with anti-FLAG M2 antibody (F1804, Sigma-Aldrich) and then with Dynabeads™ Proteine G (ThermoFisher Scientific) for 4 h. Beads were then extensively washed in modified RIPA lysis buffer and eluted with Laemmli buffer. Eluted proteins were then immuno-detected for pY^416^c-Src by western blot. Input protein extracts were immuno-blotted for NTRK3 (FLAG), pY^416^c-Src, c-Src, and GAPDH). The ImageJ software was used for band intensity quantification. Uncropped western blot images are shown in supplementary data.

### Immunohistofluorescence

The TrkC^tm1a^ is a mouse gene-trap model with a trapping cassette located in between the 4th and 5th exons of Ntrk3 gene, resulting in a full *Ntrk3* knock-out model. This model is hosted in the controlled environment of the animal facility (P-PAC) in the Cancer Research Center of Lyon, with ad libitum feeding (French Ministry for Research, registered number C2EA15, protocol CECCAPP CLB_2010_22). TrkC^tm1a^ E18.5 embryos were obtained by crossing TrkC^+/−^ mice. TrkC^tm1a^ E18.5 embryos were obtained by crossing TrkC^+^^/−^ mice. Pregnant mice were killed by cervical dislocation. Embryos were retrieved and fixated in PBS containing 4% PFA for 24 h. Fixated embryos were incubated in 30% sucrose and then embedded in OCT media (Cellpath, Newtown, UK), before being frozen at −80 °C. Embryos were PCR sexed using the mRbm31xy primers (described in [63]) and genotyped for *Ntrk3* KO using the Lar primers [36] (Supplementary table 2). Female embryos were cryosectioned (15 µm thickness cuts). Cryosections were post-fixated in PBS-PFA 4% for 15 min, blocked with 10% Goat serum diluted in PBS for 1 h and then incubated overnight at 4 °C with anti-LHβ antibody (gift of Dr J.F. Roser, University of California Davis, USA). Invitrogen Alexa Fluor antibodies were used as secondary antibodies. Nuclei were stained with DAPI and slides were mounted in ProLong Gold Antifade Mountant (ThermoFisher Scientific) for imaging. Images were acquired using the ZEISS LSM 700 confocal microscope on the Functional and Adaptive Biology Unit microscopy facility. For each genotypes, at least 6 different pituitary sections of 3 embryos were studied. To reduce position bias, images were taken at standardized anatomical positions. Only images with complete pituitary glands of similar total area along with *pars nervosa* and median eminence were kept for analysis. More than 800 LHβ positive cells where counted per genotype. To compare cell distributions between genotypes, gonadotrope cell positions were plotted on a Cartesian coordinate system with an origin point fixed at the ventro-posterior tips of the *pars distalis* (schematic representation, Fig. 7B). Cell histogram distributions were plotted on both the X and Y axis and compared between genotypes using *χ*^2^ comparison test.

### Statistical analysis

Analyses were performed in double-blinded experimental setups. Statistical analyses were performed using the Prism-GraphPad software. Test types and parameters are detailed in each figure legend.

### Representative image adjustment

Maximum displayed value of signal (ImageJ software) were linearly adjusted using identical parameters between test and control conditions.

### Supplementary information

Supplementary Information legends

Movie M1

Movie M2

Supplementary Table 1

Supplementary Table 2

Figure S1 FluoroBlok transwell migration assay.

Figure S2 Subcellular localization of total Cortactin in WT and ΔΔ cells.

Figure S3 FAK expression in WT and ΔΔ cells.

Figure S4 Mobility rescue assays.

Uncropped Western Blots

## Data Availability

The GSM3579940 and GSM1306336 NEURDO1 ChIP-seq raw data Bed files are available in the ChIP-atlas (https://chip-atlas.org/) repository. The GSE104513 αT3-1 RNA-seq dataset is available in the Gene Expression Omnibus repository and the αT3-1 ATAC-seq dataset is accessible upon reasonable request.
