# Supplementary material for: The Neurod1/4-Ntrk3-Src pathway regulates gonadotrope cell adhesion and motility
Source: Cell Death Discov. 2023 Sep 1;9:327. doi: 10.1038/s41420-023-01615-7 (PMC10474047; doi:10.1038/s41420-023-01615-7)
Supplement: Supplementary file 4 — Supplementary Table 1 [file 41420_2023_1615_MOESM4_ESM.pdf]

ID	Term	Pvalue	% Genes	Associated Genes Found
140	Steroid hormone biosynthesis	0,00	5,43	[Cyp7b1, Hsd11b2, Hsd17b12, Srd5a1, Srd5a3]
4115	p53 signaling pathway	0,00	61,11	[Aifm2, Apaf1, Atm, Bax, Bbc3, Bcl2l1, Casp3, Ccnb1, Ccnb2, Ccnd3, Ccne1, Ccne2, Ccng1, Ccng2, Cd82, Cdk1, Cdk2, Cdk4, Cdkn1a, Cdkn2a, Chek1, Ei24, Gadd45a, Gadd45b, Gadd45g, Gorab, Gtse1, Igfbp3, Mdm2, Mdm4, Perp, Pten, Rchy1, Rprm, Rrm2, Rrm2b, Sesn1, Sesn2, Sesn3, Sfn, Shisa5, Siva1, Trp53, Zmat3]
4120	Ubiquitin mediated proteolysis	0,00	64,14	[Anapc1, Anapc11, Anapc13, Anapc4, Anapc5, Anapc7, Birc2, Birc6, Brca1, Btrc, Cblb, Cdc16, Cdc20, Cdc23, Cdc34, Cul2, Cul4a, Cul5, Cul7, Ddb1, Det1, Fancl, Fbxo2, Fbxo4, Fbxw11, Fbxw7, Fbxw8, Fzr1, Herc1, Herc2, Herc3, Keap1, Klhl13, Klhl9, Map3k1, Mdm2, Mgrn1, Mid1, Nedd4, Nedd4l, Nhlrc1, Pias1, Pias3, Pias4, Ppil2, Prpf19, Rbx1, Rchy1, Rhobtb1, Rnf7, Skp1a, Skp2, Smurf1, Socs1, Socs3, Stub1, Syvn1, Traf6, Trim32, Trim37, Trip12, Uba2, Uba6, Ubb, Ubc, Ube2b, Ube2c, Ube2d1, Ube2d3, Ube2e1, Ube2e2, Ube2e3, Ube2f, Ube2g1, Ube2g2, Ube2h, Ube2i, Ube2j2, Ube2k, Ube2l6, Ube2n, Ube2o, Ube2q1, Ube2s, Ube2u, Ube2w, Ube2z, Ube3a, Ube3c, Ube4a, Ube4b, Ubox5, Wwp1]
4136	Autophagy	0,00	75,00	[Atg10, Atg12, Atg13, Atg16l1, Atg2a, Atg3, Atg4a, Atg4b, Atg4c, Atg4d, Atg5, Atg9b, Becn1, Gabarapl1, Gabarapl2, Mlst8, Mtor, Pik3c3, Ppp2ca, Ppp2cb, Rptor, Ulk2, Wipi1, Wipi2]
4137	Mitophagy	0,00	69,70	[Ambra1, Atf4, Atg5, Atg9b, Bcl2l1, Bcl2l13, Becn1, Bnip3, Bnip3l, Cited2, Csnk2a1, Csnk2a2, E2f1, Eif2ak3, Fis1, Foxo3, Fundc1, Gabarapl1, Gabarapl2, Hif1a, Jun, Mapk10, Mapk8, Mapk9, Mfn1, Mras, Nbr1, Optn, Pgam5, Pink1, Rab7, Rela, Rhot1, Rras2, Sp1, Sqstm1, Src, Tbc1d15, Tbc1d17, Trp53, Ubb, Ubc, Ulk1, Usp15, Usp30, Usp8]
4140	Autophagy	0,00	70,29	Atg5, Atg9b, Bad, Bcl2l1, Becn1, Bnip3, C9orf72, Camkk2, Cflar, Ctsd, Dapk1, Dapk3, Ddit4, Eif2ak3, Ern1, Gabarapl1, Gabarapl2, Hif1a, Igf1r, Irs1, Irs2, Irs4, Itpr1, Lamp1, Lamp2, Map2k1, Map2k2, Map3k7, Mapk1, Mapk10, Mapk3, Mapk8, Mapk9, Mlst8, Mras, Mtmr14, Mtmr4, Mtor, Nrbf2, Pdpk1, Pik3c3, Pik3ca, Pik3cb, Pik3r1, Pik3r2, Pik3r3, Ppp2ca, Ppp2cb, Prkaa1, Prkaa2, Prkaca, Prkcd, Pten, Rab33b, Rab39b, Rab7, Rab8a, Raf1, Rb1cc1, Rps6kb1, Rps6kb2, Rptor, Rraga, Rragb, Rragc, Rragd, Rras2, Sh3glb1, Snap29, Sqstm1, Stk11, Tank, Traf6, Trp53inp2, Tsc1, Ulk1, Ulk2, Uvrag, Wdr41, Wipi1, Wipi2]
4141	Protein processing in endoplasmic reticul	0,00	70,35	Hspbp1, Hsph1, Hyou1, Lman1, Man1a, Man1c1, Map2k7, Map3k5, Mapk10, Mapk8, Mapk9, Mbtps1, Mbtps2, Mogs, Nfe2l2, Nploc4, Nsfl1c, Os9, P4hb, Pdia3, Pdia4, Pdia6, Plaa, Ppp1r15a, Preb, Prkcsh, Rad23a, Rad23b, Rbx1, Rnf5, Rpn1, Rpn2, Rrbp1, Sar1a, Sar1b, Sec13, Sec23a, Sec23b, Sec24a, Sec24b, Sec24c, Sec24d, Sec31a, Sec61a1, Sec61a2, Sec61b, Sec61g, Sec62, Sel1l, Sil1, Skp1a, Ssr1, Ssr2, Ssr3, Stt3a, Stt3b, Stub1, Svip, Syvn1, Traf2, Tram1, Tram1l1, Tusc3, Ube2d1, Ube2d3, Ube2g1, Ube2g2, Ube2j2, Ube4b, Ubqln1, Ubqln4, Ubxn1, Uggt1, Uggt2, Vcp, Wfs1, Xbp1, Yod1]
4142	Lysosome	0,00	57,25	[Abca2, Abcb9, Acp2, Aga, Ap1b1, Ap1g1, Ap1m1, Ap1s1, Ap1s2, Ap3b1, Ap3b2, Ap3d1, Ap3m1, Ap3m2, Ap3s1, Ap3s2, Ap4e1, Arsb, Asah1, Atp6ap1, Atp6v0a1, Atp6v0b, Atp6v0c, Atp6v1h, Cd164, Cd63, Clta, Cltb, Cltc, Ctns, Ctsa, Ctsd, Ctsf, Ctso, Ctsz, Dnase2a, Gaa, Galc, Galns, Gba, Gga1, Gga2, Gga3, Glb1, Gm2a, Gnptab, Gns, Gusb, Hexa, Hgsnat, Hyal2, Idua, Igf2r, Lamp1, Lamp2, Laptm4a, Laptm4b, Laptm5, Litaf, M6pr, Manba, Mcoln1, Mfsd8, Naglu, Nagpa, Neu1, Npc1, Pla2g15, Psap, Scarb2, Slc11a2, Slc17a5, Smpd1, Sort1, Sumf1]

4144 Endocytosis	0,00	54,95	[Acap2, Acap3, Actr2, Actr3, Actr3b, Agap1, Agap2, Agap3, Ap2a1, Ap2a2, Ap2b1, Ap2m1, Ap2s1, Arf1, Arf2, Arf3, Arf4, Arf5, Arf6, Arfgap1, Arfgap2, Arfgap3, Arfgef1, Arfgef2, Arpc2, Arpc3, Arpc4, Arpc5l, Arrb1, Arrb2, Asap1, Asap2, Asap3, Capza1, Capza2, Capzb, Cblb, Cdc42, Chmp2a, Chmp4b, Chmp5, Chmp6, Chmp7, Clta, Cltb, Cltc, Cyth1, Cyth2, Cyth3, Dnajc6, Dnm1, Dnm2, Dnm3, Eea1, Egfr, Ehd1, Ehd3, Ehd4, Epn1, Epn2, Epn3, Eps15, Gbf1, Git1, Git2, Grk5, Grk6, H2-D1, H2-K1, Hspa2, Hspa8, Igf1r, Igf2r, Iqsec1, Iqsec2, Kif5a, Kif5b, Kif5c, Ldlr, Ldlrap1, Mdm2, Nedd4, Nedd4l, Pard3, Pard6a, Pard6b, Pard6g, Pdcd6ip, Pip5k1a, Pip5k1c, Pip5kl1, Prkci, Prkcz, Psd, Psd3, Rab10, Rab11a, Rab11b, Rab11fip1, Rab11fip2, Rab11fip3, Rab11fip5, Rab22a, Rab35, Rab4a, Rab5b, Rab5c, Rab7, Rab8a, Rabep1, Rufy1, Sh3gl1, Sh3gl2, Sh3gl3, Sh3glb1, Sh3glb2, Smad3, Smap1, Smap2, Smurf1, Snx1, Snx12, Snx2, Snx3, Snx32, Snx4, Snx5, Spg21, Src, Stam, Stam2, Tfrc, Tgfbr1, Traf6, Tsg101, Usp8, Vps25, Vps26b, Vps28, Vps29, Vps37a, Vps37b, Vps37c, Vps45, Vps4a, Vps4b, Wasl, Wipf2, Wwp1, Zfyve16]
4150 mTOR signaling pathway	0,00	58,33	Ddit4, Depdc5, Dvl3, Eif4b, Eif4e, Eif4e2, Eif4ebp1, Flcn, Fnip2, Fzd10, Fzd2, Fzd5, Fzd8, Grb10, Grb2, Gsk3b, Igf1r, Ikbkb, Insr, Irs1, Lpin1, Lpin2, Lpin3, Lrp5, Map2k1, Map2k2, Mapk1, Mapk3, Mapkap1, Mios, Mlst8, Mtor, Pdpk1, Pik3ca, Pik3cb, Pik3r1, Pik3r2, Pik3r3, Prkaa1, Prkaa2, Prkca, Prkcb, Prr5, Pten, Raf1, Rictor, Rps6ka1, Rps6ka2, Rps6ka3, Rps6kb1, Rps6kb2, Rptor, Rraga, Rragb, Rragc, Rragd, Sec13, Seh1l, Sesn2, Sgk1, Skp2, Slc38a9, Slc3a2, Slc7a5, Sos1, Sos2, Stk11, Stradb, Tbc1d7, Telo2, Tsc1, Ulk1, Ulk2, Wdr24, Wdr59, Wnt5b, Wnt9a]
590 Arachidonic acid metabolism	0,00	6,98	[Alox5, Cyp2u1, Ggt5, Pla2g6, Ptges2, Ptges3]
4010 MAPK signaling pathway	0,00	47,62	Map3k1, Map3k11, Map3k12, Map3k13, Map3k14, Map3k2, Map3k3, Map3k4, Map3k5, Map3k7, Map4k1, Map4k3, Map4k4, Mapk1, Mapk10, Mapk12, Mapk14, Mapk3, Mapk7, Mapk8, Mapk8ip1, Mapk8ip2, Mapk9, Mapkapk5, Mapt, Max, Mknk2, Mras, Nfatc3, Nfkb2, Ngfr, Nlk, Nr4a1, Pak1, Pak2, Pgf, Ppm1a, Ppp3ca, Ppp3cb, Ppp3r1, Ppp5c, Prkaca, Prkca, Prkcb, Ptpn5, Rac1, Raf1, Rap1b, Rapgef2, Rasa1, Rasa2, Rasgrf1, Rela, Rps6ka1, Rps6ka2, Rps6ka3, Rps6ka5, Rras2, Sos1, Sos2, Stk4, Stmn1, Tab1, Tab2, Taok1, Taok2, Taok3, Tgfa, Tgfbr1, Traf2, Traf6, Trp53, Vegfa]
4360 Axon guidance	0,00	54,14	[Abl1, Ablim2, Ablim3, Arhgef12, Bmpr1b, Bmpr2, Camk2b, Camk2d, Cdc42, Cdk5, Cfl2, Cxcl12, Dpysl2, Efna2, Efna3, Efna5, Efnb2, Epha7, Ephb1, Ephb2, Ephb6, Fyn, Gnai2, Gnai3, Gsk3b, Itgb1, Limk1, Limk2, Lrig2, Lrrc4, Mapk1, Mapk3, Myl12b, Nck1, Nck2, Neo1, Nfatc3, Nrp1, Ntn4, Pak1, Pak2, Pak3, Pak4, Pak7, Pard3, Pard6a, Pard6b, Pard6g, Pdpk1, Pik3ca, Pik3cb, Pik3r1, Pik3r2, Pik3r3, Plxna1, Plxna2, Plxna3, Plxnc1, Ppp3ca, Ppp3cb, Ppp3r1, Prkca, Prkcz, Ptch1, Ptk2, Rac1, Raf1, Rasa1, Rgma, Rgs3, Rnd1, Robo1, Rock1, Rock2, Sema3e, Sema3f, Sema3g, Sema4b, Sema4c, Sema4d, Sema4f, Sema4g, Sema5a, Sema5b, Sema6a, Sema6b, Sema6c, Sema6d, Slit2, Smo, Src, Srgap2, Srgap3, Ssh1, Ssh2, Ssh3, Unc5a, Wnt5b]

4514	Cell adhesion molecules	0,00	18,97	[Alcam, Cadm1, Cadm3, Cd276, Cd99l2, Cdh2, Cdh4, Cldn23, Cldn9, Cntn1, Cntnap1, Cntnap2, H2-D1, H2-K1, Itga6, Itga9, Itgav, Itgb1, Jam3, Lrrc4, Mpzl1, Ncam1, Ncam2, Negr1, Neo1, Nfasc, Nrcam, Nrxn1, Nrxn2, Ptprf, Pvr, Sdc3, Sdc4]
4530	Tight junction	0,00	55,09	[illegible] Arhgap17, Arhgef18, Arpc2, Arpc3, Arpc4, Arpc5l, Cdc42, Cdk4, Cgn, Cgnl1, Cldn23, Cldn9, Cttn, Dlg1, Dlg3, Ezr, Hspa4, Itgb1, Jam3, Jun, Llgl2, Map2k7, Map3k1, Map3k5, Mapk10, Mapk8, Mapk9, Marveld2, Mpdz, Mpp5, Msn, Myh10, Myh14, Myh9, Myl12b, Myl6b, Nedd4, Nedd4l, Nf2, Pard3, Pard6a, Pard6b, Pard6g, Pcna, Ppp2ca, Ppp2cb, Ppp2r1a, Ppp2r1b, Ppp2r2b, Ppp2r2c, Ppp2r2d, Prkaa1, Prkaa2, Prkab1, Prkab2, Prkaca, Prkag1, Prkag2, Prkce, Prkci, Prkcz, Rab8a, Rac1, Rap2c, Rapgef2, Rapgef6, Rock1, Rock2, Scrib, Slc9a3r1, Src, Stk11, Synpo, Tiam1, Tjp1, Tjp2, Tjp3, Tuba1a, Tuba1b, Tuba1c, Tuba4a, Tuba8, Vasp, Whamm]
4610	Complement and coagulation cascades	0,00	6,45	[Clu, Cr1l, F2r, Plat, Pros1, Serping1]
4640	Hematopoietic cell lineage	0,00	9,57	[Cr1l, Flt3l, Gp1bb, Il4ra, Itga2, Itga2b, Itga6, Kit, Tfrc]
830	Retinol metabolism	0,00	6,19	[Adh5, Cyp2s1, Dgat1, Dhrs3, Rdh10, Rdh13]
4728	Dopaminergic synapse	0,00	56,30	[Adcy5, Akt1, Akt2, Akt3, Arntl, Arrb1, Arrb2, Atf2, Atf4, Atf6b, Cacna1a, Cacna1b, Cacna1c, Calm1, Calm2, Calm3, Calml4, Caly, Camk2b, Camk2d, Creb1, Creb3l1, Creb3l2, Fos, Gnai2, Gnai3, Gnaq, Gnas, Gnb1, Gnb2, Gnb3, Gnb4, Gnb5, Gng10, Gng12, Gng13, Gng2, Gng3, Gng4, Gng7, Gria1, Gria2, Gria4, Gsk3a, Gsk3b, Itpr1, Kcnj3, Kcnj9, Kif5a, Kif5b, Kif5c, Mapk10, Mapk12, Mapk14, Mapk8, Mapk9, Plcb4, Ppp1ca, Ppp1cb, Ppp2ca, Ppp2cb, Ppp2r1a, Ppp2r1b, Ppp2r2b, Ppp2r2c, Ppp2r2d, Ppp2r3a, Ppp2r5a, Ppp2r5b, Ppp2r5c, Ppp2r5e, Ppp3ca, Ppp3cb, Prkaca, Prkca, Prkcb]
4740	Olfactory transduction	0,00	1,20	[Arrb1, Arrb2, Calm1, Calm2, Calm3, Calml4, Camk2b, Camk2d, Gnb1, Gng13, Gng7, Ncald, Prkaca, Slc8a1]
4742	Taste transduction	0,00	10,87	[Adcy6, Cacna1a, Cacna1c, Gnb3, Gng13, Hcn4, Htr1d, Plcb4, Prkaca, Scn3a]
4060	Cytokine-cytokine receptor interaction	0,00	9,93	[Acvr1, Acvr2a, Acvr2b, Acvrl1, Bmp8a, Bmpr1a, Bmpr1b, Bmpr2, Ccl25, Ccl27a, Ccr10, Cntf, Crlf2, Cxcl12, Gdf11, Ifnar1, Ifnar2, Il10rb, Il17ra, Il4ra, Il6st, Inhbb, Lifr, Ltbr, Ngfr, Relt, Tgfbr1, Tnfrsf18, Tnfrsf21]
3013	RNA transport	0,00	54,89	Ddx20, Eef1a1, Eef1a2, Eif1ax, Eif1b, Eif2b1, Eif2b3, Eif2b4, Eif2b5, Eif2s3x, Eif2s3y, Eif3a, Eif3b, Eif3d, Eif3e, Eif3f, Eif3g, Eif3h, Eif4a1, Eif4a2, Eif4a3, Eif4b, Eif4e, Eif4e2, Eif4ebp1, Eif4ebp2, Eif4g1, Eif4g2, Eif4g3, Eif5, Eif5b, Elac1, Elac2, Fus, Gemin5, Gemin7, Gle1, Kpnb1, Magoh, Nup133, Nup155, Nup160, Nup188, Nup205, Nup210, Nup214, Nup35, Nup37, Nup43, Nup50, Nup54, Nup85, Nup93, Nup98, Nupl1, Nupl2, Nxt1, Pabpc1, Pabpc1l, Pabpc4, Paip1, Pnn, Pop4, Pop5, Prmt5, Rae1, Ranbp2, Rangap1, Rbm8a, Rnps1, Rpp21, Sap18, Sec13, Seh1l, Senp2, Smn1, Snupn, Srrm1, Sumo2, Sumo3, Tacc3, Tardbp, Thoc1, Thoc3, Thoc5, Tpr, Trnt1, Ube2i, Upf1, Upf2, Upf3a, Upf3b, Xpo1, Xpot]

5132 Salmonella infection	0,00	56,92	Casp7, Cdc42, Chuk, Ctnnb1, Cyfip1, Cyfip2, Cyth1, Cyth2, Cyth3, Dctn1, Dctn2, Dctn3, Dctn4, Dctn5, Dctn6, Dnm2, Dync1i2, Dync1li1, Dync1li2, Dync2h1, Dync2li1, Dynll1, Dynll2, Dynlrb1, Dynlt1b, Dynlt3, Elmo1, Elmo2, Exoc2, Exoc4, Exoc7, Fadd, Fbxo22, Flna, Flnb, Fos, Fyco1, Gapdh, Gcc2, Gsdmd, Hsp90aa1, Hsp90ab1, Hsp90b1, Ikbkb, Jun, Kif5a, Kif5b, Kif5c, Klc2, Klc3, Kpna1, Kpna3, Ly96, M6pr, Map2k1, Map2k2, Map2k6, Map2k7, Map3k7, Mapk1, Mapk10, Mapk12, Mapk14, Mapk3, Mapk8, Mapk9, Myl12b, Mylpf, Myo6, Naip2, Nckap1, Nfkbia, Pak1, Pak3, Pfn2, Pik3c2a, Pik3c3, Pik3ca, Pik3cb, Pkn1, Plekhm1, Podxl, Rab5b, Rab5c, Rab7, Rab9b, Rac1, Raf1, Rala, Rela, Rhob, Ripk1, Rock2, Skp1a, Snx18, Snx33, Tab1, Tab2, Tlr2, Traf2, Traf6, Tuba1a, Tuba1b, Tuba1c, Tuba4a, Tuba8, Tubb2a, Tubb3, Tubb5, Tubb6, Txn1, Txn2, Vps16, Vps18, Vps33a, Vps39, Vps41, Wasf3, Wasl]
3015 mRNA surveillance pathway	0,00	57,84	Cstf3, Ddx19a, Ddx19b, Eif4a3, Etf1, Fus, Gle1, Gspt2, Hbs1l, Magoh, Msi1, Nudt21, Nxt1, Pabpc1, Pabpc1l, Pabpc4, Pabpn1, Papola, Papolg, Pcf11, Pelo, Pnn, Ppp1ca, Ppp1cb, Ppp2ca, Ppp2cb, Ppp2r1a, Ppp2r1b, Ppp2r2b, Ppp2r2c, Ppp2r2d, Ppp2r3a, Ppp2r5a, Ppp2r5b, Ppp2r5c, Ppp2r5e, Rbm8a, Rngtt, Rnps1, Sap18, Smg5, Smg6, Smg7, Srrm1, Ssu72, Tardbp, Upf1, Upf2, Upf3a, Upf3b, Wdr33]
5144 Malaria	0,00	7,02	[Cr1l, Lrp1, Thbs3, Tlr2]
5204 Chemical carcinogenesis	0,00	9,90	[Adh5, Aldh3b1, Arnt, Ephx1, Gstm4, Gstm5, Gsto2, Gstp1, Gstt2, Mgst3]
3018 RNA degradation	0,00	63,10	[Btg1, Btg2, Cnot1, Cnot10, Cnot2, Cnot3, Cnot6, Cnot6l, Cnot7, Cnot8, Dcp1a, Dcp1b, Dcp2, Dcps, Ddx6, Dhx36, Dis3l, Edc4, Eno1, Exosc1, Exosc2, Exosc3, Exosc4, Exosc5, Exosc6, Exosc8, Exosc9, Hspa9, Hspd1, Lsm2, Lsm3, Lsm4, Lsm5, Lsm6, Mtrex, Nudt16, Pabpc1, Pabpc1l, Pabpc4, Pan2, Parn, Patl1, Pfkl, Pfkm, Pnpt1, Tent4a, Tob1, Tob2, Ttc37, Wdr61, Xrn1, Xrn2, Zcchc7]
4061 Viral protein interaction with cytokine and	0,00	7,37	[Ccl25, Ccl27a, Ccr10, Cxcl12, Il10rb, Il6st, Ltbr]
5322 Systemic lupus erythematosus	0,00	10,81	[Actn1, Actn4, H2ac10, H2ax, H2az1, H2az2, H2bc18, H2bc4, H2bu2, H3f3a, H3f3b, H4c9, Macroh2a1, Macroh2a2, Snrpb, Snrpd1]
3040 Spliceosome	0,00	61,19	[Acin1, Bcas2, Ccdc12, Cdc40, Cdc5l, Cherp, Ctnnbl1, Cwc15, Ddx23, Ddx42, Ddx5, Dhx16, Dhx38, Dhx8, Eftud2, Eif4a3, Fus, Hnrnpc, Hnrnpu, Hspa2, Hspa8, Isy1, Lsm2, Lsm3, Lsm4, Lsm5, Lsm6, Magoh, Pcbp1, Phf5a, Ppil1, Prpf18, Prpf19, Prpf3, Prpf38a, Prpf38b, Prpf4, Prpf40a, Prpf40b, Prpf6, Prpf8, Puf60, Rbm17, Rbm25, Rbm8a, Rp9, Sf3a1, Sf3a3, Sf3b1, Sf3b2, Sf3b5, Slu7, Smndc1, Snrnp200, Snrnp27, Snrnp70, Snrpa, Snrpa1, Snrpb, Snrpb2, Snrpc, Snrpd1, Snrpe, Snrpf, Snrpg, Snw1, Srsf1, Srsf10, Srsf2, Srsf3, Srsf4, Srsf5, Srsf7, Srsf9, Syf2, Tcerg1, Thoc1, Thoc3, Tra2a, Tra2b, U2af1, Zmat2]
4068 FoxO signaling pathway	0,00	63,36	[Agap2, Akt1, Akt2, Akt3, Araf, Atg12, Atm, Bcl2l11, Bcl6, Bnip3, Cat, Ccnb1, Ccnb2, Ccng2, Cdk2, Cdkn1a, Cdkn1b, Cdkn2b, Chuk, Crebbp, Egfr, Ep300, Fbxo32, Foxo3, Foxo6, G6pc3, Gabarapl1, Gabarapl2, Gadd45a, Gadd45b, Gadd45g, Grb2, Homer1, Igf1r, Ikbkb, Insr, Irs1, Irs2, Irs4, Klf2, Map2k1, Map2k2, Mapk1, Mapk10, Mapk12, Mapk14, Mapk3, Mapk8, Mapk9, Mdm2, Nlk, Pdpk1, Pik3ca, Pik3cb, Pik3r1, Pik3r2, Pik3r3, Plk1, Plk2, Plk4, Prkaa1, Prkaa2, Prkab1, Prkab2, Prkag1, Prkag2, Prmt1, Pten, Raf1, Rbl2, Setd7, Sgk1, Sgk3, Sirt1, Skp2, Smad3, Sod2, Sos1, Sos2, Stat3, Stk11, Stk4, Tgfbr1]

4070 Phosphatidylinositol signaling system	0,00	57,29	[Calm1, Calm2, Calm3, Calml4, Cdipt, Cds1, Cds2, Dgka, Dgkd, Dgkh, Dgki, Dgkz, Impa2, Impad1, Inpp1, Inpp4a, Inpp5a, Inpp5b, Inpp5e, Inpp5f, Inppl1, Ip6k2, Ipmk, Ippk, Itpkc, Itpr1, Mtmr1, Mtmr14, Mtmr2, Mtmr4, Mtmr6, Mtmr7, Pi4k2b, Pi4kb, Pik3c2a, Pik3c3, Pik3ca, Pik3cb, Pik3r1, Pik3r2, Pik3r3, Pikfyve, Pip4k2a, Pip4k2c, Pip4p1, Pip5k1a, Pip5k1c, Plcb4, Ppip5k2, Prkca, Prkcb, Pten, Sacm1l, Synj1, Synj2]
4080 Neuroactive ligand-receptor interaction	0,00	9,50	[Adcyap1r1, Adra2a, Adrb1, Chrm4, Chrna1, Chrnb2, F2r, Gabrb3, Gabrd, Gabrg2, Glrb, Gria1, Gria2, Gria4, Grik3, Grin1, Grin2d, Hcrt, Hrh3, Htr1d, Kiss1, Lhcgr, Lpar2, Lpar6, Npff, Npy, Nr3c1, Oxtr, P2rx4, Pard3, Ptger4, Sct, Tacr1, Thra]
4110 Cell cycle	0,00	68,29	Anapc7, Atm, Ccna2, Ccnb1, Ccnb2, Ccnd3, Ccne1, Ccne2, Ccnh, Cdc14a, Cdc14b, Cdc16, Cdc20, Cdc23, Cdc25a, Cdc25b, Cdc45, Cdc6, Cdc7, Cdk1, Cdk2, Cdk4, Cdk7, Cdkn1a, Cdkn1b, Cdkn1c, Cdkn2a, Cdkn2b, Chek1, Crebbp, E2f1, E2f2, E2f3, E2f4, Ep300, Espl1, Fzr1, Gadd45a, Gadd45b, Gadd45g, Gsk3b, Hdac1, Hdac2, Mad1l1, Mad2l1, Mad2l2, Mcm2, Mcm3, Mcm4, Mcm5, Mcm6, Mcm7, Mdm2, Pcna, Pkmyt1, Plk1, Rad21, Rb1, Rbl1, Rbl2, Rbx1, Sfn, Skp1a, Skp2, Smad3, Smc3, Stag1, Stag2, Tfdp1, Tfdp2, Trp53, Ttk, Wee1, Ywhab, Ywhae, Ywhah, Ywhaq, Ywhaz]
3030 DNA replication	0,00	80,00	[Fen1, Lig1, Mcm2, Mcm3, Mcm4, Mcm5, Mcm6, Mcm7, Pcna, Pola2, Pold1, Pold2, Pold3, Pold4, Pole2, Pole4, Prim1, Prim2, Rfc1, Rfc2, Rfc3, Rfc4, Rfc5, Rnaseh1, Rnaseh2b, Rnaseh2c, Rpa2, Ssbp1]
3430 Mismatch repair	0,00	81,82	[Lig1, Mlh1, Mlh3, Msh2, Msh6, Pcna, Pms2, Pold1, Pold2, Pold3, Pold4, Rfc1, Rfc2, Rfc3, Rfc4, Rfc5, Rpa2, Ssbp1]
4114 Oocyte meiosis	0,00	58,82	[Adcy1, Adcy5, Adcy6, Adcy7, Adcy9, Anapc1, Anapc11, Anapc13, Anapc4, Anapc5, Anapc7, Btrc, Calm1, Calm2, Calm3, Calml4, Camk2b, Camk2d, Ccnb1, Ccnb2, Ccne1, Ccne2, Cdc16, Cdc20, Cdc23, Cdk1, Cdk2, Cpeb2, Cpeb3, Cpeb4, Espl1, Fbxo5, Fbxw11, Igf1r, Itpr1, Mad1l1, Mad2l1, Mad2l2, Map2k1, Mapk1, Mapk12, Mapk14, Mapk3, Pkmyt1, Plk1, Ppp1ca, Ppp1cb, Ppp2ca, Ppp2cb, Ppp2r1a, Ppp2r1b, Ppp2r5a, Ppp2r5b, Ppp2r5c, Ppp2r5e, Ppp3ca, Ppp3cb, Ppp3r1, Prkaca, Rbx1, Rps6ka1, Rps6ka2, Rps6ka3, Skp1a, Smc3, Ywhab, Ywhae, Ywhah, Ywhaq, Ywhaz]
4914 Progesterone-mediated oocyte maturatior	0,00	64,44	[Adcy1, Adcy5, Adcy6, Adcy7, Adcy9, Akt1, Akt2, Akt3, Anapc1, Anapc11, Anapc13, Anapc4, Anapc5, Anapc7, Araf, Ccna2, Ccnb1, Ccnb2, Cdc16, Cdc23, Cdc25a, Cdc25b, Cdk1, Cdk2, Cpeb2, Cpeb3, Cpeb4, Fzr1, Gnai2, Gnai3, Hsp90aa1, Hsp90ab1, Igf1r, Kif22, Mad1l1, Mad2l1, Mad2l2, Map2k1, Mapk1, Mapk10, Mapk12, Mapk14, Mapk3, Mapk8, Mapk9, Pik3ca, Pik3cb, Pik3r1, Pik3r2, Pik3r3, Pkmyt1, Plk1, Prkaca, Raf1, Rps6ka1, Rps6ka2, Rps6ka3, Stk10]
4666 Fc gamma R-mediated phagocytosis	0,00	57,61	[Actr2, Actr3, Actr3b, Akt1, Akt2, Akt3, Arf6, Arpc2, Arpc3, Arpc4, Arpc5l, Asap1, Asap2, Asap3, Cdc42, Cfl2, Crk, Crkl, Dnm2, Dock1, Gab2, Gsn, Inppl1, Limk1, Limk2, Map2k1, Mapk1, Mapk3, Marcks, Marcksl1, Pak1, Pik3ca, Pik3cb, Pik3r1, Pik3r2, Pik3r3, Pip5k1a, Pip5k1c, Pla2g6, Prkca, Prkcb, Prkcd, Prkce, Rac1, Raf1, Rps6kb1, Rps6kb2, Vasp, Vav2, Vav3, Wasf1, Wasf2, Wasf3]

5135 Yersinia infection	0,00	57,46	[Actb, Actr2, Actr3, Actr3b, Akt1, Akt2, Akt3, Arf6, Arhgef1, Arhgef12, Arhgef7, Arpc2, Arpc3, Arpc4, Arpc5l, Baiap2, Bcar1, Cdc42, Chuk, Crk, Crkl, Dock1, Elmo1, Elmo2, Fos, Git2, Gnaq, Gsk3b, Ikbkb, Irf3, Itgb1, Jun, Limk1, Map2k1, Map2k2, Map2k6, Map2k7, Map3k7, Mapk1, Mapk10, Mapk12, Mapk14, Mapk3, Mapk8, Mapk9, Nfatc3, Nfkbia, Pik3ca, Pik3cb, Pik3r1, Pik3r2, Pik3r3, Pip5k1a, Pip5k1c, Pkn1, Pkn2, Ptk2, Ptk2b, Pxn, Rac1, Rela, Rock1, Rock2, Rps6ka1, Rps6ka2, Rps6ka3, Skap2, Src, Tab1, Tab2, Traf2, Traf6, Vav2, Vav3, Wasf2, Wasl, Wipf2]
510 N-Glycan biosynthesis	0,00	74,00	[Alg1, Alg10b, Alg11, Alg12, Alg13, Alg14, Alg2, Alg5, Alg6, Alg9, B4galt1, B4galt3, Dad1, Ddost, Dolk, Dolpp1, Dpagt1, Dpm1, Fut8, Ganab, Glt28d2, Man1a, Man1c1, Man2a1, Mgat1, Mgat3, Mgat4a, Mgat4b, Mgat5, Mogs, Rpn1, Rpn2, Srd5a3, St6gal1, Stt3a, Stt3b, Tusc3]
513 Various types of N-glycan biosynthesis	0,00	75,00	[Alg1, Alg11, Alg12, Alg13, Alg14, Alg2, Alg9, B4galnt4, B4galt1, B4galt3, Chst8, Chst9, Dad1, Ddost, Fut8, Glt28d2, Hexa, Hexdc, Man1a, Man1c1, Man2a1, Mgat1, Mgat4a, Mgat4b, Rpn1, Rpn2, St3gal3, Stt3a, Stt3b, Tusc3]
4152 AMPK signaling pathway	0,00	60,32	[Acacb, Adipor1, Adipor2, Akt1, Akt2, Akt3, Cab39, Camkk2, Ccna2, Cpt1a, Cpt1c, Creb1, Creb3l1, Creb3l2, Eef2k, Eif4ebp1, Fasn, Foxo3, G6pc3, Gys1, Hmgcr, Igf1r, Insr, Irs1, Irs2, Irs4, Lipe, Map3k7, Mlycd, Mtor, Pdpk1, Pfkfb2, Pfkfb3, Pfkl, Pfkm, Pik3ca, Pik3cb, Pik3r1, Pik3r2, Pik3r3, Ppp2ca, Ppp2cb, Ppp2r1a, Ppp2r1b, Ppp2r2b, Ppp2r2c, Ppp2r2d, Ppp2r3a, Ppp2r5a, Ppp2r5b, Ppp2r5c, Ppp2r5e, Prkaa1, Prkaa2, Prkab1, Prkab2, Prkag1, Prkag2, Rab10, Rab11b, Rab14, Rab2a, Rab8a, Rps6kb1, Rps6kb2, Rptor, Scd1, Scd2, Scd3, Sirt1, Srebf1, Stk11, Stradb, Tbc1d1, Tsc1, Ulk1]
4211 Longevity regulating pathway	0,00	66,67	[Adcy1, Adcy5, Adcy6, Adcy7, Adcy9, Adipor1, Adipor2, Akt1, Akt2, Akt3, Appl1, Atf2, Atf4, Atf6b, Atg13, Atg5, Bax, Camk4, Camkk2, Cat, Creb1, Creb3l1, Creb3l2, Ehmt1, Eif4e, Eif4e2, Eif4ebp1, Foxo3, Igf1r, Insr, Irs1, Irs2, Irs4, Mtor, Pik3ca, Pik3cb, Pik3r1, Pik3r2, Pik3r3, Prkaa1, Prkaa2, Prkab1, Prkab2, Prkaca, Prkag1, Prkag2, Rb1cc1, Rela, Rps6kb1, Rps6kb2, Rptor, Sesn1, Sesn2, Sesn3, Sirt1, Sod2, Stk11, Trp53, Tsc1, Ulk1]
4213 Longevity regulating pathway	0,00	66,13	[Adcy1, Adcy5, Adcy6, Adcy7, Adcy9, Akt1, Akt2, Akt3, Atg5, Cat, Clpb, Eif4ebp2, Foxo3, Hdac1, Hdac2, Hspa2, Hspa8, Igf1r, Insr, Irs1, Irs2, Irs4, Mtor, Pik3ca, Pik3cb, Pik3r1, Pik3r2, Pik3r3, Prkaa1, Prkaa2, Prkab1, Prkab2, Prkaca, Prkag1, Prkag2, Rps6kb1, Rps6kb2, Rptor, Sirt1, Sod1, Sod2]
4714 Thermogenesis	0,00	53,25	Mgll, Mlst8, Mtor, Ndufa10, Ndufa12, Ndufa13, Ndufa2, Ndufa3, Ndufa4, Ndufa4l2, Ndufa5, Ndufa6, Ndufa7, Ndufa9, Ndufab1, Ndufaf1, Ndufaf3, Ndufaf4, Ndufb2, Ndufb3, Ndufb4, Ndufb5, Ndufb7, Ndufb9, Ndufc1, Ndufc2, Ndufs2, Ndufs3, Ndufs4, Ndufs6, Ndufs7, Ndufs8, Ndufv1, Npr1, Plin1, Pnpla2, Prkaa1, Prkaa2, Prkab1, Prkab2, Prkaca, Prkag1, Prkag2, Rps6ka1, Rps6ka2, Rps6ka3, Rps6kb1, Rps6kb2, Rptor, Sdha, Sirt6, Slc25a29, Smarca2, Smarca4, Smarcb1, Smarcc1, Smarcc2, Smarcd1, Smarcd2, Smarcd3, Smarce1, Sos1, Sos2, Tsc1, Uqcr10, Uqcr11, Uqcrc1, Uqcrfs1, Uqcrq, Zfp516]

4932 Non-alcoholic fatty liver disease	0,00	57,62	[illegible] Casp3, Casp7, Cdc42, Cox5a, Cox5b, Cox6c, Cox7a2, Cox7a2l, Cox8a, Cyc1, Ddit3, Eif2ak3, Ern1, Fos, Gsk3a, Gsk3b, Ikbkb, Insr, Irs1, Irs2, Jun, Map3k11, Map3k5, Mapk10, Mapk8, Mapk9, Mlxip, Mlxipl, Ndufa10, Ndufa12, Ndufa13, Ndufa2, Ndufa3, Ndufa4, Ndufa4l2, Ndufa5, Ndufa6, Ndufa7, Ndufa9, Ndufab1, Ndufb2, Ndufb3, Ndufb4, Ndufb5, Ndufb7, Ndufb9, Ndufc1, Ndufc2, Ndufs2, Ndufs3, Ndufs4, Ndufs6, Ndufs7, Ndufs8, Ndufv1, Pik3ca, Pik3cb, Pik3r1, Pik3r2, Pik3r3, Prkaa1, Prkaa2, Prkab1, Prkab2, Prkag1, Prkag2, Rac1, Rela, Rxra, Sdha, Socs3, Srebf1, Traf2, Uqcr10, Uqcr11, Uqcrc1, Uqcrfs1, Uqcrq, Xbp1]
5010 Alzheimer disease	0,00	52,03	Cacna1c, Calm1, Calm2, Calm3, Calml4, Capn1, Capn2, Casp3, Casp7, Cdk5, Chuk, Cox5a, Cox5b, Cox6c, Cox7a2, Cox7a2l, Cox8a, Csnk1a1, Csnk2a1, Csnk2a2, Ctnnb1, Cyc1, Ddit3, Dvl3, Eif2ak3, Ern1, Fadd, Frat1, Frat2, Fzd10, Fzd2, Fzd5, Fzd8, Gapdh, Gnaq, Grin1, Grin2d, Gsk3b, Hsd17b10, Ikbkb, Insr, Irs1, Irs2, Irs4, Itpr1, Kif5a, Kif5b, Kif5c, Klc2, Klc3, Lpl, Lrp1, Lrp5, Map2k1, Map2k2, Map2k7, Map3k5, Mapk1, Mapk10, Mapk3, Mapk8, Mapk9, Mapt, Mtor, Ncstn, Ndufa10, Ndufa12, Ndufa13, Ndufa2, Ndufa3, Ndufa4, Ndufa4l2, Ndufa5, Ndufa6, Ndufa7, Ndufa9, Ndufab1, Ndufb2, Ndufb3, Ndufb4, Ndufb5, Ndufb7, Ndufb9, Ndufc1, Ndufc2, Ndufs2, Ndufs3, Ndufs4, Ndufs6, Ndufs7, Ndufs8, Ndufv1, Nrbf2, Pik3c3, Pik3ca, Pik3cb, Pik3r1, Pik3r2, Pik3r3, Plcb4, Ppp3ca, Ppp3cb, Ppp3r1, Psen1, Psen2, Psma1, Psma3, Psma5, Psma6, Psmb1, Psmb2, Psmb3, Psmb6, Psmb7, Psmc1, Psmc2, Psmc3, Psmc6, Psmd1, Psmd12, Psmd14, Psmd2, Psmd3, Psmd4, Psmd6, Psmd8, Psmd9, Raf1, Rb1cc1, Rela, Rtn3, Rtn4, Sdha, Slc25a4, Slc25a5, Snca, Traf2, Tuba1a, Tuba1b, Tuba1c, Tuba4a, Tuba8, Tubb2a, Tubb3, Tubb5, Tubb6, Ulk1, Ulk2, Uqcr10, Uqcr11, Uqcrc1, Uqcrfs1, Uqcrq, Vdac1, Vdac2, Vdac3, Wipi1, Wipi2, Wnt5b, Wnt9a, Xbp1]
5012 Parkinson disease	0,00	55,06	[Adcy5, Adrm1, Apaf1, Atf4, Atf6, Atp5b, Atp5e, Atp5g1, Atp5g3, Atp5h, Atp5j, Atp5o, Atp5pb, Bax, Bcl2l1, Calm1, Calm2, Calm3, Calml4, Camk2b, Camk2d, Casp3, Cox5a, Cox5b, Cox6c, Cox7a2, Cox7a2l, Cox8a, Cyc1, Ddit3, Dusp1, Eif2ak3, Ern1, Gnai2, Gnai3, Gnas, Hspa5, Htra2, Itpr1, Kif5a, Kif5b, Kif5c, Klc2, Klc3, Map3k5, Mapk10, Mapk8, Mapk9, Mapt, Mfn1, Ndufa10, Ndufa12, Ndufa13, Ndufa2, Ndufa3, Ndufa4, Ndufa4l2, Ndufa5, Ndufa6, Ndufa7, Ndufa9, Ndufab1, Ndufb2, Ndufb3, Ndufb4, Ndufb5, Ndufb7, Ndufb9, Ndufc1, Ndufc2, Ndufs2, Ndufs3, Ndufs4, Ndufs6, Ndufs7, Ndufs8, Ndufv1, Nfe2l2, Park7, Pink1, Prkaca, Psma1, Psma3, Psma5, Psma6, Psmb1, Psmb2, Psmb3, Psmb6, Psmb7, Psmc1, Psmc2, Psmc3, Psmc6, Psmd1, Psmd12, Psmd14, Psmd2, Psmd3, Psmd4, Psmd6, Psmd8, Psmd9, Sdha, Slc25a4, Slc25a5, Snca, Trap1, Trp53, Tuba1a, Tuba1b, Tuba1c, Tuba4a, Tuba8, Tubb2a, Tubb3, Tubb5, Tubb6, Txn1, Txn2, Ubb, Ubc, Ube2g1, Ube2g2, Ube2j2, Ube2l6, Uchl1, Uqcr10, Uqcr11, Uqcrc1, Uqcrfs1, Uqcrq, Vdac1, Vdac2, Vdac3, Xbp1]
5014 Amyotrophic lateral sclerosis	0,00	53,39	Cox5b, Cox6c, Cox7a2, Cox7a2l, Cox8a, Cyc1, Dctn1, Dctn2, Dctn3, Dctn4, Dctn5, Dctn6, Ddit3, Derl1, Eif2ak3, Erbb4, Ern1, Fig4, Fus, Gabarapl1, Gabarapl2, Gle1, Gria1, Gria2, Grin1, Grin2d, Hap1, Hnrnpa2b1, Hspa5, Kif5a, Kif5b, Kif5c, Klc2, Klc3, Map2k6, Map3k5, Mapk12, Mapk14, Matr3, Mtor, Ndufa10, Ndufa12, Ndufa13, Ndufa2, Ndufa3, Ndufa4, Ndufa4l2, Ndufa5, Ndufa6, Ndufa7, Ndufa9, Ndufab1, Ndufb2, Ndufb3, Ndufb4, Ndufb5, Ndufb7, Ndufb9, Ndufc1, Ndufc2, Ndufs2, Ndufs3, Ndufs4, Ndufs6, Ndufs7, Ndufs8, Ndufv1, Nefl, Nefm, Nrbf2, Nrg1, Nup133, Nup155, Nup160, Nup188, Nup205, Nup210, Nup214, Nup35, Nup37, Nup43, Nup50, Nup54, Nup85, Nup93, Nup98, Nupl1, Nxt1, Optn, Pfn2, Pik3c3, Pink1, Ppp3ca, Ppp3cb, Ppp3r1, Psma1, Psma3, Psma5, Psma6, Psmb1, Psmb2, Psmb3, Psmb6, Psmb7, Psmc1, Psmc2, Psmc3, Psmc6, Psmd1, Psmd12, Psmd14, Psmd2, Psmd3, Psmd4, Psmd6, Psmd8, Psmd9, Rab39b, Rab8a, Rac1, Rae1, Ranbp2, Rb1cc1, Sdha, Sec13, Seh1l, Setx, Sigmar1, Slc1a2, Sod1, Spg11, Sqstm1, Srsf3, Srsf7, Tank, Tardbp, Tomm40, Tomm40l, Tpr, Traf2, Trp53, Tuba1a, Tuba1b, Tuba1c, Tuba4a, Tuba8, Tubb2a, Tubb3, Tubb5, Tubb6, Ubqln1, Ubqln4, Ulk1, Ulk2, Uqcr10, Uqcr11, Uqcrc1, Uqcrfs1, Uqcrq, Vapb, Vcp, Vdac1, Wdr41, Wipi1, Wipi2, Xbp1]

5016 Huntington disease	0,00	56,62	Atp5e, Atp5g1, Atp5g3, Atp5h, Atp5j, Atp5o, Atp5pb, Bax, Bbc3, Bdnf, Becn1, Cacna1b, Casp3, Clta, Cltb, Cltc, Cox5a, Cox5b, Cox6c, Cox7a2, Cox7a2l, Cox8a, Creb1, Creb3l1, Creb3l2, Crebbp, Cyc1, Dctn1, Dctn2, Dctn3, Dctn4, Dctn5, Dctn6, Dlg4, Ep300, Ern1, Gnaq, Gria1, Gria2, Gria4, Grin1, Hap1, Hdac1, Hdac2, Hip1, Htt, Ift57, Itpr1, Kif5a, Kif5b, Kif5c, Klc2, Klc3, Map2k7, Map3k10, Map3k5, Mapk10, Mapk8, Mapk9, Mtor, Ndufa10, Ndufa12, Ndufa13, Ndufa2, Ndufa3, Ndufa4, Ndufa4l2, Ndufa5, Ndufa6, Ndufa7, Ndufa9, Ndufab1, Ndufb2, Ndufb3, Ndufb4, Ndufb5, Ndufb7, Ndufb9, Ndufc1, Ndufc2, Ndufs2, Ndufs3, Ndufs4, Ndufs6, Ndufs7, Ndufs8, Ndufv1, Nrbf2, Nrf1, Pik3c3, Plcb4, Polr2a, Polr2e, Polr2h, Polr2i, Polr2j, Polr2k, Psma1, Psma3, Psma5, Psma6, Psmb1, Psmb2, Psmb3, Psmb6, Psmb7, Psmc1, Psmc2, Psmc3, Psmc6, Psmd1, Psmd12, Psmd14, Psmd2, Psmd3, Psmd4, Psmd6, Psmd8, Psmd9, Rb1cc1, Rcor1, Sdha, Sin3a, Slc1a2, Slc25a4, Slc25a5, Sod1, Sod2, Sp1, Stx1a, Tbp, Tbpl1, Tfam, Traf2, Trp53, Tuba1a, Tuba1b, Tuba1c, Tuba4a, Tuba8, Tubb2a, Tubb3, Tubb5, Tubb6, Ulk1, Ulk2, Uqcr10, Uqcr11, Uqcrc1, Uqcrfs1, Uqcrq, Vdac1, Vdac2, Vdac3, Wipi1, Wipi2]
5017 Spinocerebellar ataxia	0,00	60,99	Atg2a, Atp2a2, Atp2a3, Atxn10, Atxn3, Becn1, Cacna1a, Cic, Dab1, Ern1, Gnaq, Gria1, Gria2, Grin1, Grin2d, Gtf2b, Itpr1, Map3k5, Mapk10, Mapk8, Mapk9, Mtor, Nop56, Nrbf2, Oma1, Opa1, Pik3c3, Pik3ca, Pik3cb, Pik3r1, Pik3r2, Pik3r3, Plcb4, Prkca, Prkcb, Psma1, Psma3, Psma5, Psma6, Psmb1, Psmb2, Psmb3, Psmb6, Psmb7, Psmc1, Psmc2, Psmc3, Psmc6, Psmd1, Psmd12, Psmd14, Psmd2, Psmd3, Psmd4, Psmd6, Psmd8, Psmd9, Pum1, Pum2, Rb1cc1, Rbpj, Reln, Rora, Slc25a4, Slc25a5, Sp1, Tbp, Tbpl1, Traf2, Ulk1, Ulk2, Vdac1, Vdac2, Vdac3, Vldlr, Wipi1, Wipi2, Xbp1]
5020 Prion disease	0,00	51,49	[illegible] Cox8a, Creb1, Creb3l1, Creb3l2, Csnk2a1, Csnk2a2, Cyc1, Ddit3, Egr1, Eif2ak3, Fyn, Grin1, Grin2d, Gsk3b, Hspa2, Hspa5, Hspa8, Itpr1, Kif5a, Kif5b, Kif5c, Klc2, Klc3, Lamc1, Mapk1, Mapk10, Mapk12, Mapk14, Mapk3, Mapk8, Mapk9, Ncam1, Ncam2, Ndufa10, Ndufa12, Ndufa13, Ndufa2, Ndufa3, Ndufa4, Ndufa4l2, Ndufa5, Ndufa6, Ndufa7, Ndufa9, Ndufab1, Ndufb2, Ndufb3, Ndufb4, Ndufb5, Ndufb7, Ndufb9, Ndufc1, Ndufc2, Ndufs2, Ndufs3, Ndufs4, Ndufs6, Ndufs7, Ndufs8, Ndufv1, Pik3ca, Pik3cb, Pik3r1, Pik3r2, Pik3r3, Ppp3ca, Ppp3cb, Ppp3r1, Prkaca, Prkcd, Psma1, Psma3, Psma5, Psma6, Psmb1, Psmb2, Psmb3, Psmb6, Psmb7, Psmc1, Psmc2, Psmc3, Psmc6, Psmd1, Psmd12, Psmd14, Psmd2, Psmd3, Psmd4, Psmd6, Psmd8, Psmd9, Rac1, Sdha, Slc25a4, Slc25a5, Sod1, Stip1, Tuba1a, Tuba1b, Tuba1c, Tuba4a, Tuba8, Tubb2a, Tubb3, Tubb5, Tubb6, Uqcr10, Uqcr11, Uqcrc1, Uqcrfs1, Uqcrq, Vdac1, Vdac2, Vdac3]
5022 Pathways of neurodegeneration	0,00	50,21	Camk2b, Camk2d, Capn1, Capn2, Casp3, Casp7, Cat, Cdk5, Cox5a, Cox5b, Cox6c, Cox7a2, Cox7a2l, Cox8a, Csnk1a1, Csnk2a1, Csnk2a2, Ctnnb1, Cyc1, Dctn1, Dctn2, Dctn3, Dctn4, Dctn5, Dctn6, Ddit3, Derl1, Dlg4, Dvl3, Eif2ak3, Ern1, Fadd, Fig4, Frat1, Frat2, Fus, Fzd10, Fzd2, Fzd5, Fzd8, Gabarapl1, Gabarapl2, Gnaq, Gria1, Gria2, Gria4, Grin1, Grin2d, Gsk3b, Hap1, Hip1, Hsd17b10, Hspa5, Htra2, Htt, Ift57, Itpr1, Kif5a, Kif5b, Kif5c, Klc2, Klc3, Lrp5, Map2k1, Map2k2, Map2k6, Map2k7, Map3k10, Map3k5, Mapk1, Mapk10, Mapk12, Mapk14, Mapk3, Mapk8, Mapk9, Mapt, Mfn1, Mtor, Ndufa10, Ndufa12, Ndufa13, Ndufa2, Ndufa3, Ndufa4, Ndufa4l2, Ndufa5, Ndufa6, Ndufa7, Ndufa9, Ndufab1, Ndufb2, Ndufb3, Ndufb4, Ndufb5, Ndufb7, Ndufb9, Ndufc1, Ndufc2, Ndufs2, Ndufs3, Ndufs4, Ndufs6, Ndufs7, Ndufs8, Ndufv1, Nefl, Nefm, Nrbf2, Optn, Park7, Pik3c3, Pink1, Plcb4, Ppp3ca, Ppp3cb, Ppp3r1, Prkca, Prkcb, Psen1, Psen2, Psma1, Psma3, Psma5, Psma6, Psmb1, Psmb2, Psmb3, Psmb6, Psmb7, Psmc1, Psmc2, Psmc3, Psmc6, Psmd1, Psmd12, Psmd14, Psmd2, Psmd3, Psmd4, Psmd6, Psmd8, Psmd9, Rab39b, Rab8a, Rac1, Raf1, Rb1cc1, Rela, Sdha, Sigmar1, Slc25a4, Slc25a5, Snca, Sod1, Spg11, Sqstm1, Stx1a, Tank, Tardbp, Tomm40, Tomm40l, Traf2, Trap1, Tuba1a, Tuba1b, Tuba1c, Tuba4a, Tuba8, Tubb2a, Tubb3, Tubb5, Tubb6, Ubb, Ubc, Ube2g1, Ube2g2, Ube2j2, Ube2l6, Uchl1, Ulk1, Ulk2, Uqcr10, Uqcr11, Uqcrc1, Uqcrfs1, Uqcrq, Vapb, Vcp, Vdac1, Vdac2, Vdac3, Wdr41, Wipi1, Wipi2, Wnt5b, Wnt9a, Xbp1]

4012 ErbB signaling pathway	0,00	71,43	[illegible] Cblb, Cdkn1a, Cdkn1b, Crk, Crkl, Egfr, Eif4ebp1, Erbb3, Erbb4, Gab1, Grb2, Gsk3b, Hbegf, Jun, Map2k1, Map2k2, Map2k7, Mapk1, Mapk10, Mapk3, Mapk8, Mapk9, Mtor, Nck1, Nck2, Nrg1, Pak1, Pak2, Pak3, Pak4, Pak7, Pik3ca, Pik3cb, Pik3r1, Pik3r2, Pik3r3, Prkca, Prkcb, Ptk2, Raf1, Rps6kb1, Rps6kb2, Shc1, Shc2, Shc3, Sos1, Sos2, Src, Stat5a, Stat5b, Tgfa]
4210 Apoptosis	0,00	55,88	[Actb, Akt1, Akt2, Akt3, Apaf1, Atf4, Atm, Bad, Bax, Bbc3, Bcl2l1, Bcl2l11, Birc2, Birc5, Capn1, Capn2, Casp3, Casp6, Casp7, Cflar, Chuk, Ctsd, Ctsf, Ctso, Ctsz, Dab2ip, Ddit3, Dffb, Diablo, Eif2ak3, Endog, Ern1, Fadd, Fos, Gadd45a, Gadd45b, Gadd45g, Hrk, Htra2, Ikbkb, Itpr1, Jun, Lmna, Lmnb1, Lmnb2, Map2k1, Map2k2, Map3k14, Map3k5, Mapk1, Mapk10, Mapk3, Mapk8, Mapk9, Mcl1, Nfkbia, Parp1, Parp2, Parp3, Parp4, Pdpk1, Pik3ca, Pik3cb, Pik3r1, Pik3r2, Pik3r3, Raf1, Rela, Ripk1, Traf2, Trp53, Tuba1a, Tuba1b, Tuba1c, Tuba4a, Tuba8]
4218 Cellular senescence	0,00	51,09	Ccne2, Cdc25a, Cdk1, Cdk2, Cdk4, Cdkn1a, Cdkn2a, Cdkn2b, Chek1, E2f1, E2f2, E2f3, E2f4, Eif4ebp1, Fbxw11, Foxm1, Foxo3, Gadd45a, Gadd45b, Gadd45g, H2-D1, H2-K1, Hipk1, Hipk3, Hus1, Igfbp3, Itpr1, Lin37, Lin52, Map2k1, Map2k2, Map2k6, Mapk1, Mapk12, Mapk14, Mapk3, Mdm2, Mras, Mre11a, Mtor, Mybl2, Nbn, Nfatc3, Pik3ca, Pik3cb, Pik3r1, Pik3r2, Pik3r3, Ppp1ca, Ppp1cb, Ppp3ca, Ppp3cb, Ppp3r1, Pten, Rad1, Rad50, Raf1, Rassf5, Rb1, Rbbp4, Rbl1, Rbl2, Rela, Rras2, Sirt1, Slc25a4, Slc25a5, Smad3, Sqstm1, Tgfbr1, Traf3ip2, Trp53, Trpm7, Tsc1, Vdac1, Vdac2, Vdac3, Zfp36l2]
4510 **Focal adhesion**	0,00	50,25	Col4a2, Crk, Crkl, Ctnnb1, Dock1, Egfr, Flna, Flnb, Fyn, Grb2, Gsk3b, Igf1r, Itga2, Itga2b, Itga6, Itga9, Itgav, Itgb1, Itgb5, Jun, Kdr, Lama1, Lama5, Lamb1, Lamb2, Lamb3, Lamc1, Map2k1, Mapk1, Mapk10, Mapk3, Mapk8, Mapk9, Myl12b, Mylpf, Pak1, Pak2, Pak3, Pak4, Pak7, Parva, Parvb, Pdpk1, Pgf, Pik3ca, Pik3cb, Pik3r1, Pik3r2, Pik3r3, Pip5k1a, Pip5k1c, Ppp1ca, Ppp1cb, Ppp1r12a, Ppp1r12b, Ppp1r12c, Prkca, Prkcb, Pten, Ptk2, Pxn, Rac1, Raf1, Rap1b, Rapgef1, Rasgrf1, Reln, Rock1, Rock2, Shc1, Shc2, Shc3, Sos1, Sos2, Spp1, Src, Thbs3, Tln1, Tln2, Tnr, Vasp, Vav2, Vav3, Vegfa, Zyx]
4722 Neurotrophin signaling pathway	0,00	65,29	[Abl1, Akt1, Akt2, Akt3, Arhgdia, Atf4, Bad, Bax, Bdnf, Calm1, Calm2, Calm3, Calml4, Camk2b, Camk2d, Camk4, Cdc42, Crk, Crkl, Foxo3, Gab1, Grb2, Gsk3b, Ikbkb, Irs1, Jun, Kidins220, Maged1, Map2k1, Map2k2, Map2k5, Map2k7, Map3k1, Map3k3, Map3k5, Mapk1, Mapk10, Mapk12, Mapk14, Mapk3, Mapk7, Mapk8, Mapk9, Matk, Nfkbia, Nfkbib, Ngfr, Ntrk3, Pdpk1, Pik3ca, Pik3cb, Pik3r1, Pik3r2, Pik3r3, Prdm4, Prkcd, Psen1, Psen2, Rac1, Raf1, Rap1b, Rapgef1, Rela, Rps6ka1, Rps6ka2, Rps6ka3, Rps6ka5, Sh2b1, Sh2b2, Shc1, Shc2, Shc3, Sort1, Sos1, Sos2, Traf6, Trp53, Ywhae, Zfp110]
4910 Insulin signaling pathway	0,00	64,75	Calml4, Cblb, Crk, Crkl, Eif4e, Eif4e2, Eif4ebp1, Exoc7, Fasn, Flot1, G6pc3, Gck, Grb2, Gsk3b, Gys1, Hk1, Hk2, Ikbkb, Inpp5a, Inppl1, Insr, Irs1, Irs2, Irs4, Lipe, Map2k1, Map2k2, Mapk1, Mapk10, Mapk3, Mapk8, Mapk9, Mknk2, Mtor, Pdpk1, Phka1, Phka2, Phkb, Phkg2, Pik3ca, Pik3cb, Pik3r1, Pik3r2, Pik3r3, Ppp1ca, Ppp1cb, Ppp1r3a, Ppp1r3e, Prkaa1, Prkaa2, Prkab1, Prkab2, Prkaca, Prkag1, Prkag2, Prkar1a, Prkar1b, Prkar2b, Prkci, Prkcz, Ptpn1, Ptprf, Pygb, Raf1, Rapgef1, Rhoq, Rps6kb1, Rps6kb2, Rptor, Sh2b2, Shc1, Shc2, Shc3, Socs1, Socs2, Socs3, Sorbs1, Sos1, Sos2, Srebf1, Tsc1]
4931 Insulin resistance	0,00	59,09	[Acacb, Akt1, Akt2, Akt3, Cpt1a, Creb1, Creb3l1, Creb3l2, G6pc3, Gfpt1, Gsk3b, Gys1, Ikbkb, Insr, Irs1, Irs2, Mapk10, Mapk8, Mapk9, Mlxip, Mlxipl, Mtor, Nfkbia, Nr1h2, Oga, Ogt, Pdpk1, Pik3ca, Pik3cb, Pik3r1, Pik3r2, Pik3r3, Ppp1ca, Ppp1cb, Ppp1r3a, Ppp1r3e, Prkaa1, Prkaa2, Prkab1, Prkab2, Prkag1, Prkag2, Prkcb, Prkcd, Prkce, Prkcz, Pten, Ptpn1, Ptprf, Pygb, Rela, Rps6ka1, Rps6ka2, Rps6ka3, Rps6kb1, Rps6kb2, Slc27a1, Slc27a3, Slc27a4, Slc27a6, Socs3, Srebf1, Stat3, Tbc1d4, Trib3]

4935 Growth hormone synthesis, secretion and	0,00	60,34	[Adcy1, Adcy5, Adcy6, Adcy7, Adcy9, Akt1, Akt2, Akt3, Atf2, Atf4, Atf6b, Bcar1, Cacna1c, Creb1, Creb3l1, Creb3l2, Crebbp, Crk, Crkl, Ep300, Fos, Gna11, Gnai2, Gnai3, Gnaq, Gnas, Grb2, Gsk3b, Igfbp3, Irs1, Irs2, Irs4, Itpr1, Jak2, Junb, Map2k1, Map2k2, Map2k6, Map3k1, Mapk1, Mapk10, Mapk12, Mapk14, Mapk3, Mapk8, Mapk9, Mtor, Pik3ca, Pik3cb, Pik3r1, Pik3r2, Pik3r3, Plcb4, Prkaca, Prkca, Prkcb, Ptk2, Raf1, Shc1, Shc2, Shc3, Socs1, Socs2, Socs3, Sos1, Sos2, Stat1, Stat3, Stat5a, Stat5b]
5161 Hepatitis B	0,00	54,60	Bad, Bax, Birc5, Casp3, Ccna2, Ccne1, Ccne2, Cdk2, Cdkn1a, Chuk, Creb1, Creb3l1, Creb3l2, Crebbp, Ddb1, Ddx3x, E2f1, E2f2, E2f3, Ep300, Fadd, Fos, Grb2, Hspg2, Ifnar1, Ikbkb, Irf3, Irf7, Jak1, Jak2, Jun, Map2k1, Map2k2, Map2k6, Map2k7, Map3k1, Map3k7, Mapk1, Mapk10, Mapk12, Mapk14, Mapk3, Mapk8, Mapk9, Mavs, Nfatc3, Nfkbia, Pcna, Pik3ca, Pik3cb, Pik3r1, Pik3r2, Pik3r3, Prkca, Prkcb, Ptk2b, Raf1, Rb1, Rela, Smad3, Sos1, Sos2, Src, Stat1, Stat3, Stat5a, Stat5b, Stat6, Tab1, Tab2, Tgfbr1, Tlr2, Traf3, Traf6, Trp53, Tyk2, Vdac3, Ywhab, Ywhaq, Ywhaz]
5210 Colorectal cancer	0,00	59,09	[Akt1, Akt2, Akt3, Apc, Appl1, Araf, Axin2, Bad, Bax, Bbc3, Bcl2l11, Birc5, Casp3, Cdkn1a, Ctnnb1, Egfr, Fos, Gadd45a, Gadd45b, Gadd45g, Grb2, Gsk3b, Jun, Map2k1, Map2k2, Mapk1, Mapk10, Mapk3, Mapk8, Mapk9, Mlh1, Msh2, Msh6, Mtor, Pik3ca, Pik3cb, Pik3r1, Pik3r2, Pik3r3, Rac1, Raf1, Rala, Ralb, Ralgds, Rps6kb1, Rps6kb2, Smad3, Sos1, Sos2, Tgfa, Tgfbr1, Trp53]
5211 Renal cell carcinoma	0,00	63,24	[Akt1, Akt2, Akt3, Araf, Arnt, Bad, Cdc42, Cdkn1a, Crebbp, Crk, Crkl, Cul2, Egln2, Ep300, Flcn, Gab1, Grb2, Hif1a, Jun, Map2k1, Map2k2, Mapk1, Mapk3, Pak1, Pak2, Pak3, Pak4, Pak7, Pik3ca, Pik3cb, Pik3r1, Pik3r2, Pik3r3, Prcc, Rac1, Raf1, Rap1b, Rapgef1, Rbx1, Sos1, Sos2, Tgfa, Vegfa]
5212 Pancreatic cancer	0,00	68,42	[Akt1, Akt2, Akt3, Araf, Bad, Bax, Bcl2l1, Brca2, Cdc42, Cdk4, Cdkn1a, Cdkn2a, Chuk, E2f1, E2f2, E2f3, Egfr, Gadd45a, Gadd45b, Gadd45g, Ikbkb, Jak1, Map2k1, Mapk1, Mapk10, Mapk3, Mapk8, Mapk9, Mtor, Pik3ca, Pik3cb, Pik3r1, Pik3r2, Pik3r3, Rac1, Rad51, Raf1, Rala, Ralb, Ralbp1, Ralgds, Rb1, Rela, Rps6kb1, Rps6kb2, Smad3, Stat1, Stat3, Tgfa, Tgfbr1, Trp53, Vegfa]
5214 Glioma	0,00	63,51	[Akt1, Akt2, Akt3, Araf, Bax, Calm1, Calm2, Calm3, Calml4, Camk2b, Camk2d, Camk4, Cdk4, Cdkn1a, Cdkn2a, E2f1, E2f2, E2f3, Egfr, Gadd45a, Gadd45b, Gadd45g, Grb2, Igf1r, Map2k1, Map2k2, Mapk1, Mapk3, Mdm2, Mtor, Pik3ca, Pik3cb, Pik3r1, Pik3r2, Pik3r3, Prkca, Prkcb, Pten, Raf1, Rb1, Shc1, Shc2, Shc3, Sos1, Sos2, Tgfa, Trp53]
5220 Chronic myeloid leukemia	0,00	69,74	[Abl1, Akt1, Akt2, Akt3, Araf, Bad, Bax, Bcl2l1, Bcr, Cdk4, Cdkn1a, Cdkn1b, Cdkn2a, Chuk, Crk, Crkl, Ctbp1, Ctbp2, E2f1, E2f2, E2f3, Gab2, Gadd45a, Gadd45b, Gadd45g, Grb2, Hdac1, Hdac2, Ikbkb, Map2k1, Map2k2, Mapk1, Mapk3, Mdm2, Nfkbia, Pik3ca, Pik3cb, Pik3r1, Pik3r2, Pik3r3, Raf1, Rb1, Rela, Shc1, Shc2, Shc3, Smad3, Sos1, Sos2, Stat5a, Stat5b, Tgfbr1, Trp53]
5222 Small cell lung cancer	0,00	58,06	[Akt1, Akt2, Akt3, Apaf1, Bax, Bcl2l1, Birc2, Casp3, Ccne1, Ccne2, Cdk2, Cdk4, Cdkn1a, Cdkn1b, Cdkn2b, Chuk, Cks2, Col4a1, Col4a2, E2f1, E2f2, E2f3, Gadd45a, Gadd45b, Gadd45g, Ikbkb, Itga2, Itga2b, Itga6, Itgav, Itgb1, Lama1, Lama5, Lamb1, Lamb2, Lamb3, Lamc1, Max, Nfkbia, Pik3ca, Pik3cb, Pik3r1, Pik3r2, Pik3r3, Pten, Ptk2, Rb1, Rela, Rxra, Skp2, Traf2, Traf3, Traf6, Trp53]

5223 Non-small cell lung cancer	0,00	65,28	[Akt1, Akt2, Akt3, Araf, Bad, Bax, Cdk4, Cdkn1a, Cdkn2a, E2f1, E2f2, E2f3, Egfr, Eml4, Foxo3, Gadd45a, Gadd45b, Gadd45g, Grb2, Kif5a, Kif5b, Kif5c, Map2k1, Map2k2, Mapk1, Mapk3, Pdpk1, Pik3ca, Pik3cb, Pik3r1, Pik3r2, Pik3r3, Prkca, Prkcb, Raf1, Rassf1, Rassf5, Rb1, Rxra, Sos1, Sos2, Stat3, Stat5a, Stat5b, Stk4, Tgfa, Trp53]
5225 Hepatocellular carcinoma	0,00	51,72	Arid1b, Arid2, Axin2, Bad, Bax, Bcl2l1, Brd7, Cdk4, Cdkn1a, Cdkn2a, Csnk1a1, Ctnnb1, Dpf1, Dvl3, E2f1, E2f2, E2f3, Egfr, Frat1, Frat2, Fzd10, Fzd2, Fzd5, Fzd8, Gab1, Gadd45a, Gadd45b, Gadd45g, Grb2, Gsk3b, Gstm4, Gstm5, Gsto2, Gstp1, Gstt2, Hmox1, Igf1r, Keap1, Lrp5, Map2k1, Map2k2, Mapk1, Mapk3, Mgst3, Mtor, Nfe2l2, Nqo1, Pbrm1, Pik3ca, Pik3cb, Pik3r1, Pik3r2, Pik3r3, Prkca, Prkcb, Pten, Raf1, Rb1, Rps6kb1, Rps6kb2, Shc1, Shc2, Shc3, Smad3, Smarca2, Smarca4, Smarcb1, Smarcc1, Smarcc2, Smarcd1, Smarcd2, Smarcd3, Smarce1, Sos1, Sos2, Tgfa, Tgfbr1, Trp53, Txnrd3, Wnt5b, Wnt9a]
