# Supplementary material for: The Neurod1/4-Ntrk3-Src pathway regulates gonadotrope cell adhesion and motility
Source: Cell Death Discov. 2023 Sep 1;9:327. doi: 10.1038/s41420-023-01615-7 (PMC10474047; doi:10.1038/s41420-023-01615-7)
Supplement: Supplementary file 5 — Supplementary Table 2 [file 41420_2023_1615_MOESM5_ESM.pdf]

Antibodies **IF acquisition settings**

Target	antibody	Laser intensity	Digital gain
Vinculin	V9131hVIN-1 Sigma	2%	850
FAK	sc-557 Santa Cruz	2%	700
Zyxin	ABC1387 Merck	2%	750
Cortactin	05-180 Cortactin Millipor	2%	750
pY397 FAK	#8556 pY397 Cell signaling	2%	900
pY576/577 FAK	#3284 pY925 Cell signaling	2%	900
pY925 FAK	#3284 pY925 Cell signaling	2%	900
PY861 FAK	#700154 Phospho-FAK (Tyr861)	2%	700
c-Src	#2108 c-Src Cell signaling	2%	850
pY416 Scr	#2105 pY527 Cell signaling	2%	900
pY527 Scr	#2101 pY416 Cell signaling	2%	800
3XFLAG	M2 anti FLAG sigma		
Lhb	518B7 gift from Dr J.F. Roser, University of California Davis, USA	2%	650

Primers

Expression primers

qPCR NeuroD4 F1	CGGGAAAGAGAATCTATACATGG
qPCR NeuroD4 R1	CTCAGACCTTTGTCCATCCAG
qPCRmNeurod1F2	CGCAGAAGGCAAGGTGTC
qPCRmNeurod1R2	CGCTCTCGCTGTATGATTTG
RTqPCR_mCsk_F1	CGAGCAAGCTGAGCATTGATG
RTqPCR_mCsk_R1	CCATCGGCATCTGTGGTGTAG
RTqPCR_mSrcin1_F1	CATGGTCAGAAGGCAGCTCC
RTqPCR_mSrcin1_R1	AGGGCTTCTCTGTGCGGTATC
RTqPCR_ptpn13_F1	GCTCAGGAACCCTCATTTGCA
RTqPCR_ptpn13_R1	AGCGCACTAGGTCAGAGATGT
RTqPCR_ptpn1_F1	ACAGTACGACAGTTGGAGTTGG
RTqPCR_ptpn1_R1	GAGGAAAGAAGCCGGTGACTC
RTqPCR_ptpn11_F1	CAGACAAGTGGTGATCAGAGTCC
RTqPCR_ptpn11_R1	CACGTTCTCATAGACTCGGGC
RtqPRC_mPtpn12_F1	AGGAGAGTATTGCCGATGTCCA
RtqPRC_mPtpn12_R1	GGAGTTCGTTCAGGTAAAGGAGG
mRNA_Dab1F1	TGACCCCTGTGACTTCTACCA
mRNA_Dab1R1	CTGACGTGGGATGCTGATGAT
mNtrk3_RTqPCR_F1	ATCAACAAGTATGGTCGCCGG
mNtrk3_RTqPCR_R1	TGCCATGGTTGATGTGATGCA
mNtrk3_201_RtqPCR_F[illegible]	TTACTACAGGGTGGGAGGACA
mNtrk3_201_RtqPCR_R[illegible]	CTGTGGTGAACTTCCGGTACA
mEgfr_qPCR_F1	AATGTCTGCCACCTATGCCAC
mEgfr_qPCR_R1	CCAGATGGCCACACTTCACAT
RTpcrmActbF1	CCACCATGTACCCAGGCATT
RTpcrmActbR1	GAGTACTTGCGCTCAGGAGG
RT_Adcyap1r1F3	AGTCATTGCTTCGTTTCCACC
RT_Adcyap1r1R3	GCCTTCAATGAACAGCCAGAA
qPCRmAff3_F2	CTCAGAGAGCACTTCCGGC
qPCRmAff3_R2	TGTTAGAGGACGCAGGTTCG
RTqmErbb4F	CCTCGAGGAGAACTGGATGAA

RTqmErbb4R	TTCTTCCTTCGGGACACAAAA
mFyn_RTqPCR_F1	GGTCACCAAAGGAAGAGTGCC
mFyn_RTqPCR_R1	CAGCAGTGGATCATGAGCTCG
qPCRmKif26b_F	AGTACCTGATGCTGGACCCC
qPCRmKif26b_R	GCTCTGTCACACCCTCCAGT
RTqmNgr1F	ACGACTGTCACCCAGACTCCT
RTqmNgr1R	GCCTGCTGTTCTCTACCGATG
mNrp1_RtqPCR_F1	TCAGGTGGAGTGTGCTGACC
mNrp1_RtqPCR_R1	AGCTTGGGAATAGATGAAGTTGC
mRNA_Ntng2F1	TCGATGCAATGAGACAGGCTT
mRNA_Ntng2R1	GGTCATCGTCGCACACATTG
RTqmRac1F	AAGCTGACTCCCATCACCTACC
RTqmRac1R	TTGAGTCCTCGCTGTGTGAGA
mRNARelnF1	ACCGTGGACAAAGCAGTACTG
mRNARelnR1	AACTCCTTTCATCCGAGCTTC
mqpcrST18F1	TGCTGACATCCAACTTCCAC
mqpcrST18R1	GGGGAATAGTCCTGTTCCAGA
mRNA_Smoc1F2	CAAGAGCATAGAGGCCGATGA
mRNA_Smoc1R2	GCTGCTGAGTTAATGGCCTGA
mGnRHR-F	CAGCTTTCATGATGGTGGTG
mGnRHR-R	GGTCACACATTGCGAGAAGA
mSrc_qPCR_F	ACCCATTTACATTGTGACAGAGT
mSrc_qPCR_R	CTGAGCAGACATGTCCACCAG
mqpcrST18F1	TGCTGACATCCAACTTCCAC
mqpcrST18R1	GGGGAATAGTCCTGTTCCAGA
mRNA_Tnfsf12F1	GGCTGGGCTCTACTACCTGTA
mRNA_Tnfsf12R1	TGAGAATTCTTCCAGGCAGCG
mActr2_qPCR_F	TCGAGGTTGGAACGAGAGCTT
mActr2_qPCR_R	GGGTGGGTCTTCAATTCGGAT
mAkt1_qPCR_F1	ATCATGCAGCACCGGTTCTTT
mAkt1_qPCR_R1	TGGTGTCAGTCTCAGAGGTGA
mArhgef2_RTqPCR_F1	GGCGACGCTTTATACTTGAGC
mArhgef2_RTqPCR_R1	GGAACGAACAGTCACAGGCAA
mAuts2_RTqPCR_F1	TCCACTTTATTCTCTGCCGCT
mAuts2_RTqPCR_R1	GCGGCAGGGTTGAGAAAGTTA
mBbs4_RTqPCR_F1	GCCCTCTGGAATTTGACTCTGA
mBbs4_RTqPCR_R1	CGGTGCTTTGTCTTGGGATCT
mCdk5_RTqPCR_F2	CCCTGGCAATGATGTGGATGA
mCdk5_RTqPCR_R2	CCAAGGATGTTGTAGCTGGGT
mCrk_RTqPCR_F1	AATGCCTACGACAAGACAGCC
mCrk_RTqPCR_R1	GGAAGTGACCTCGTTTGCCAT
mCtnna2_RTqPCR_F1	GCAGGCTCTCGAATGGACAAA
mCtnna2_RTqPCR_R1	CAATCCGCTGAAGGTAGGCTA
mFlna_RtqPCR_F1	TGTAGGCCAGAAGAGCAACT
mFlna_RtqPCR_R1	ACCAGGATCTCTTCACAGGGT
RTqmKif20bF	GAAATTTCATCTCCCATCAACAT
RTqmKif20bF	AAGCCGTCGTTTGAGAATCTG

mMapk1_qPCR_F1	ACCTGGAGCAGTATTATGACCCA
mMapk1_qPCR_R1	GGTAAGTCGTCCAACTCCATGT
mMyh10_RTqPCR_F1	AGATTGGGCAGCTGGAGGAG
mMyh10_RTqPCR_R1	GCTCGTCTTCAACCTGCATGA
mPafah1b1_RTqPCR_F1	GCCTGGACCTTTCTTGCTATCT
mPafah1b1_RTqPCR_R1	ACAGAACTCCACGTACCCAGT
mPhactr1_RTqPCR_F1	AGATGCCCAAGACTATGACCGA
mPhactr1_RTqPCR_R1	AGACAGCAGAGCACTCAAGAG
mPten_qPCR_F1	CCACAAACAGAACAAGATGCTCA
mPten_qPCR_R1	GGTTTCCTCTGGTCCTGGTATGA
mPtk2_qPCR_F1	TCAGGCATCTCTTCTGGACCA
mPtk2_qPCR_R1	ACCTGTCCCATCCCTCGAAG
mPxn_qPCR_F1	ACATTTCAGCCCTCAACACCC
mPxn_qPCR_R1	GCCCGTCGTGCTCAAAGAAG
mTln1_qPCR_F1	ACTCTGAGGCAATGAAACGGC
mTln1_qPCR_R1	TTCTCCTGGTCTTCGAAGGCT
mSrf_RTqPCR_F1	GACAGCAGCACAGACCTCAC
mSrf_RTqPCR_R1	ACTAGGGTACATCATGTGGCC
mSrgap2_RTqPCR_F1	AGTTTCAATAACCATCGGCCCA
mSrgap2_RTqPCR_R1	GCTCATTCAGGGCAGAGTTCA
RTqPCR_ZyxF2	CCTCCACCAGCTCAAAACCAA
RTqPCR_ZyxR2	CAGCTGCTCCAACTCCTCTAC

gRNA primers

RNAg NeuroD4 R6 T1 F	CACCGAGGCAAGGGATTATCCAC
RNAg NeuroD4 R6 T1 R	AAACGTGGATAATCCCTTGCCTC
RNAg NeuroD4 R6 T2 F	CACCGACTTAGGAGACATAGTAT
RNAg NeuroD4 R6 T2 R	AAACATACTATGTCTCCTAAGTC
gRNA mNeuroD1 T1 F	CACCTACCTACTACGCAGCACCG
gRNA mNeuroD1 T1 R	AAACCGGTGCTGCGTAGTAGGTA
gRNA mNeuroD1 T3 F	CACCTCTCAGGACGAGGAACACG
gRNA mNeuroD1 T3 R	AAACCGTGTTCCTCGTCCTGAGA
CRISPRi_Ptpn13F1	CACCGGGCAGCGGCGCGGAGACCT
CRISPRi_Ptpn13R1	AAACAGGTCTCCGCGCCGCTGCCC
CRISPRi_Ptpn13F2	CACCGCCAGGAGGCTGAGGCGGAC
CRISPRi_Ptpn13R2	AAACGTCCGCCTCAGCCTCCTGGC
CRISPRi_Ptpn13F3	CACCGCGGCGAGGGTGACACACCA
CRISPRi_Ptpn13R3	AAACTGGTGTGTCACCCTCGCCGC
CRISPRi_Ptpn13F4	CACCGAGAGCCGGGGAGCGCTGCT
CRISPRi_Ptpn13R4	AAACAGCAGCGCTCCCCGGCTCTC
CRISPRi_Ptpn13F5	CACCGGGGGAGCGCTGCTCGGCAG
CRISPRi_Ptpn13R5	AAACCTGCCGAGCAGCGCTCCCCC

Genotyping primers

mNeuroD1 geno F	GAGTTGGGAGTGACTTGGGA
mNeuroD1 geno R	GGGTCTTGGAGTAGCAAGGT
mNeuroD4-R6 geno F	ATAAACAAGCTTGACTCTGGGACATTACAT

mNeuroD4-R6 geno R	CCAAGGACCGGTTAAATGTGCCCCAATACT
Lar3	CAACGGGTTCTTCTGTTAGTCC
Lar3p	CCCAACTCCCTCCCAGGACTGAGG
Lar5p	GTGGAGTACTGAAGTCCCTGTTGC
mRbm31xyF	CACCTTAAGAACAAGCCAATACA
mRbm31xyR	GGCTTGTCCTGAAAACATTTGG

Cloning primers

AflII-mNtrk3-201/202/205	ATGATGCTTAAGCCACCATGGATGTCTCTCTTTGCCCA
Xma1-mNtrk3-201/202 R	ATGATGCCCGGGTGCCAAGAATGTCCAGGTAGA
BamH1-mNtrk3-205 R	ATGATGGGATCCAAAGCCATGACGTCCTTTGC
AflII-mDab1 F	ATGATGCTTAAGCCACCATGTCAACTGAGACAGAAGTTCAAG
BamhI-mDab1 R	ATGATGGGATCCGCTACCGTCTTGTGGACTTATATTATC
Dab1-Y185/197D F	CCCACCAGCCAAAAGAAGGAAGGTGTTGATGATGTGCCAAAAAG
Dab1-Y185/197D R	CTTCTTTTGGCTGGTGGGAACCTGGTCCACAGCTTGTTCACAC
AflII-Src F	ATGATGCTTAAGCCACCATGGGCAGCAACAAGAGCAA
BamHI-Src R	ATGATGGGATCCTAGGTTCTCCCCGGGCTGGT
NheI-Src eGFP F	ATGATGGCTAGCCACCATGGGCAGCAACAAGAGCAAG
BamhI-Src eGFP F	ATGATGGGATCCCCTAGGTTCTCCCCGGGCTG
KpnI-R2-NeuroD4 F	ATGATGGGTACCTAGTAGAGTCACTAATACTACGTCC
Xho1-R2-NeuroD4 R	ATGATGCTCGAGCTCTTTCCCGGGGTGAGCTCCCGCG
Bamh1-R1-NeuroD4 F	ATGATGGGATCCTGGTCTTAATTCAACAAAGGCAAGT
SalI-R1-NeuroD4 R	ATGATGGTCGACTGTGCAATCAGCAAAAGGAGGGAGT
Bamh1-R3-NeuroD4 F	ATGATGGGATCCGTTGCTGTAGCTCTATTGATCTACA
SalI-R3-NeuroD4 R	ATGATGGTCGACTTGTTTTCATTGTAACCTCATTTAC
Bamh1-R4-NeuroD4 F	ATGATGGGATCCTCTATAGACAGTTCCTTCATAAACA
SalI-R4-NeuroD4 R	ATGATGGTCGACTTTCCATATCTGACTCTAATCCACC
Bamh1-R5-NeuroD4 F	ATGATGGGATCCCTTTAATAATTTGTAGTCATCATTG
SalI-R5-NeuroD4 R	ATGATGGTCGACGGATTCAAAGCATACACCACCATGC
Bamh1-R6-NeuroD4 F	ATGATGGGATCCACAGAGAGTAGGATTTCTGATTCT
SalI-R6-NeuroD4 R	ATGATGGTCGACCATTCTGTGTCTATATATAACT
