# Supplementary figures and images for: The Neurod1/4-Ntrk3-Src pathway regulates gonadotrope cell adhesion and motility
Source: Cell Death Discov. 2023 Sep 1;9:327. doi: 10.1038/s41420-023-01615-7 (PMC10474047; doi:10.1038/s41420-023-01615-7)

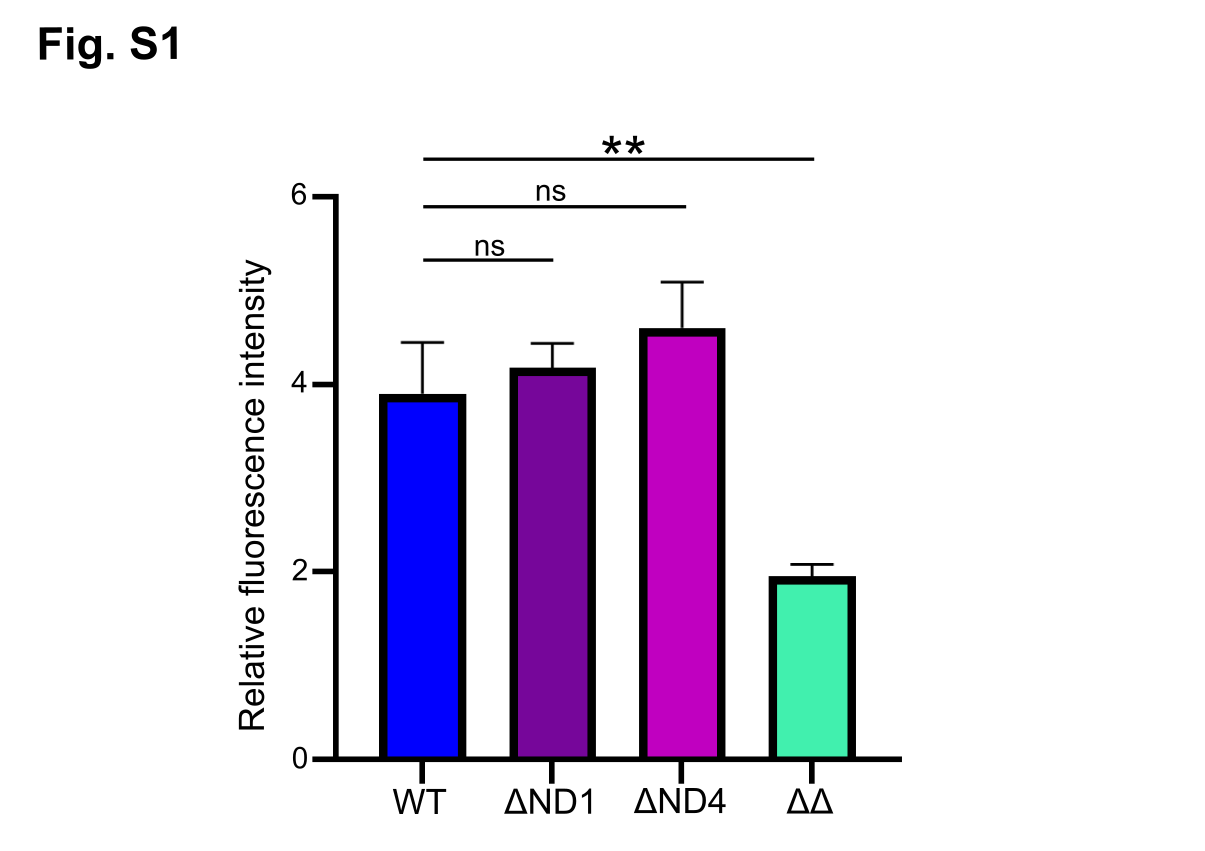

Supplement: Supplementary file 6 — Figure S1 FluoroBlok transwell migration assay. [file 41420_2023_1615_MOESM6_ESM.png]

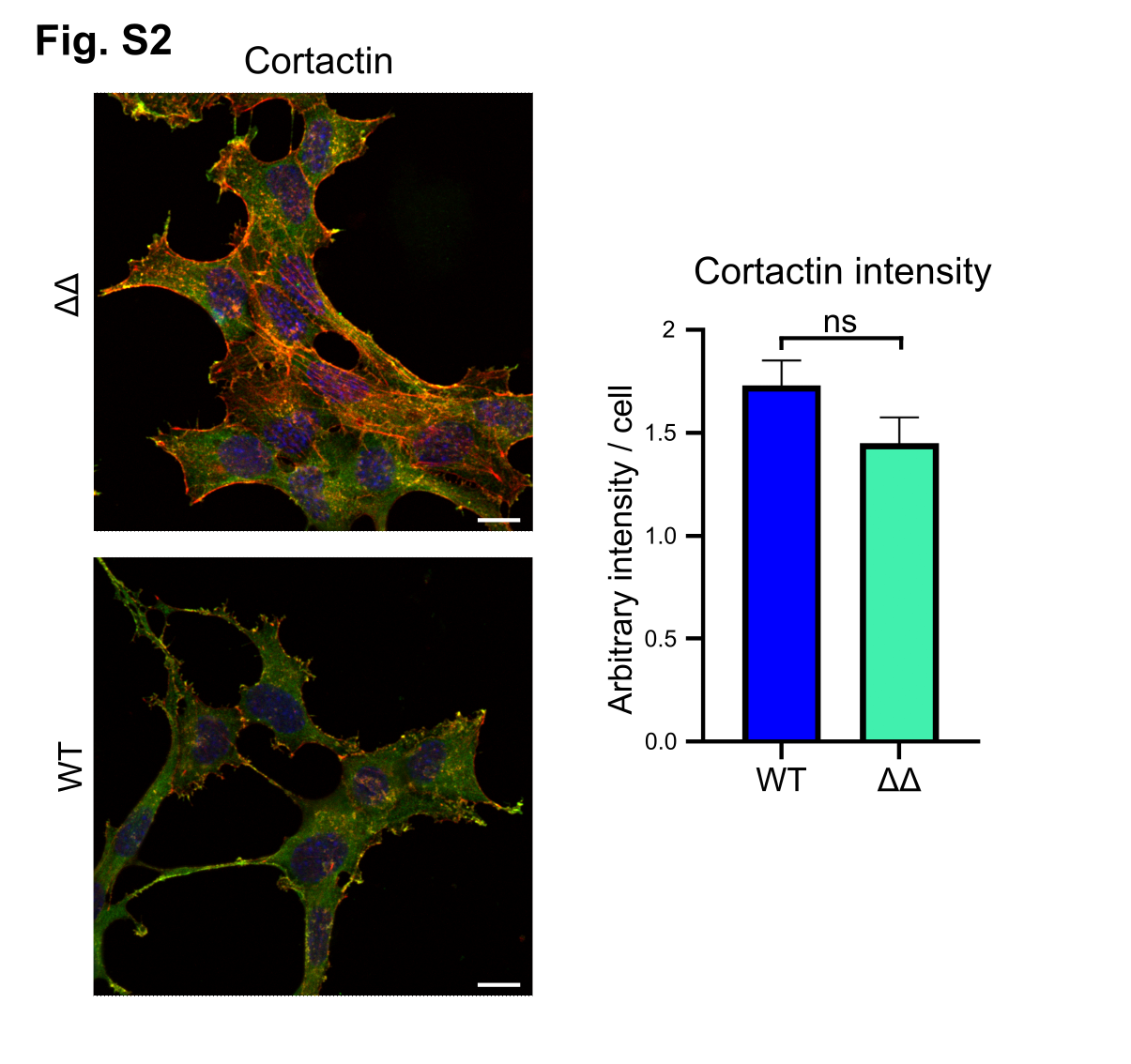

Supplement: Supplementary file 7 — Figure S2 Subcellular localization of total Cortactin in WT and ΔΔ cells. [file 41420_2023_1615_MOESM7_ESM.png]

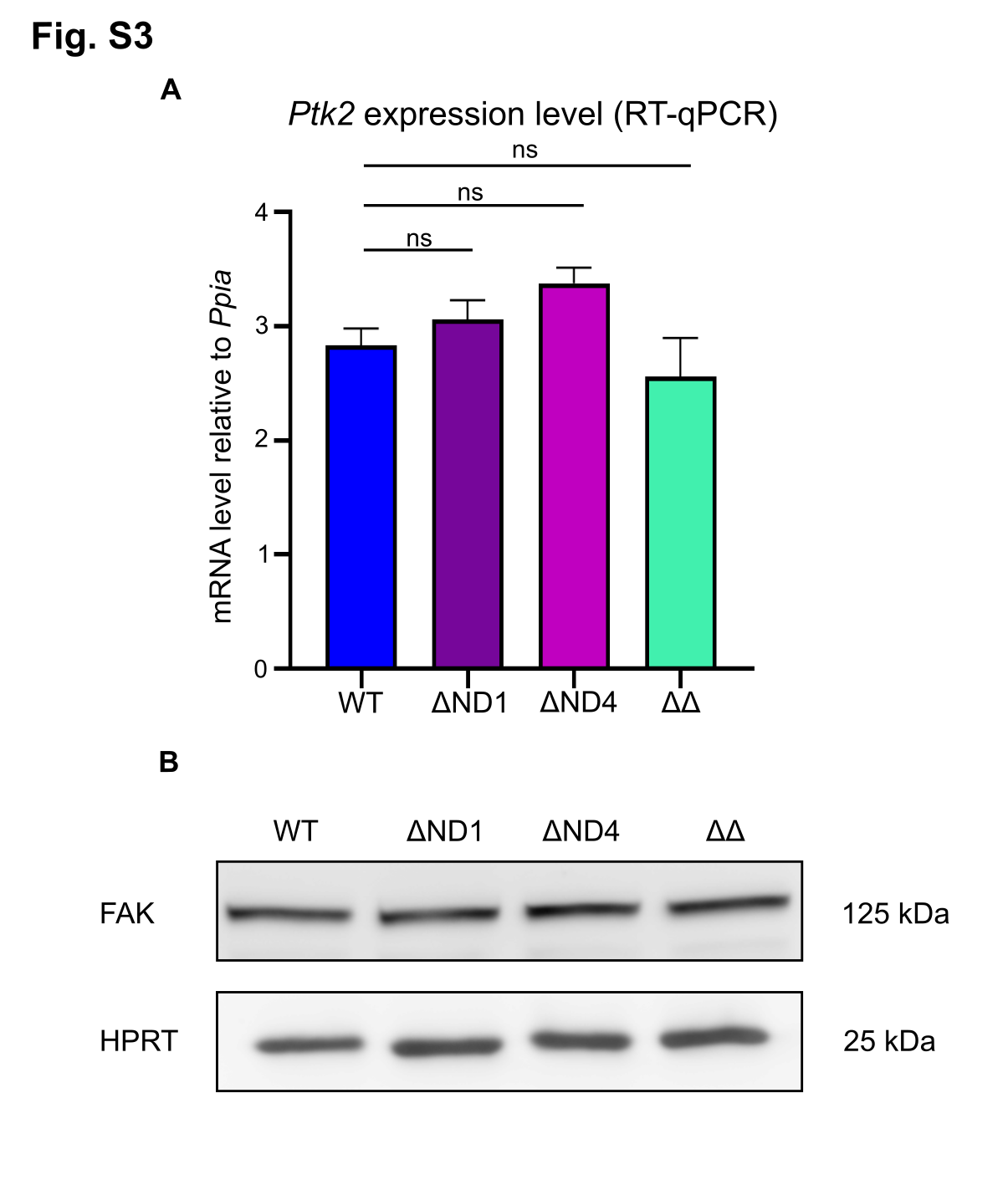

Supplement: Supplementary file 8 — Figure S3 FAK expression in WT and ΔΔ cells. [file 41420_2023_1615_MOESM8_ESM.png]

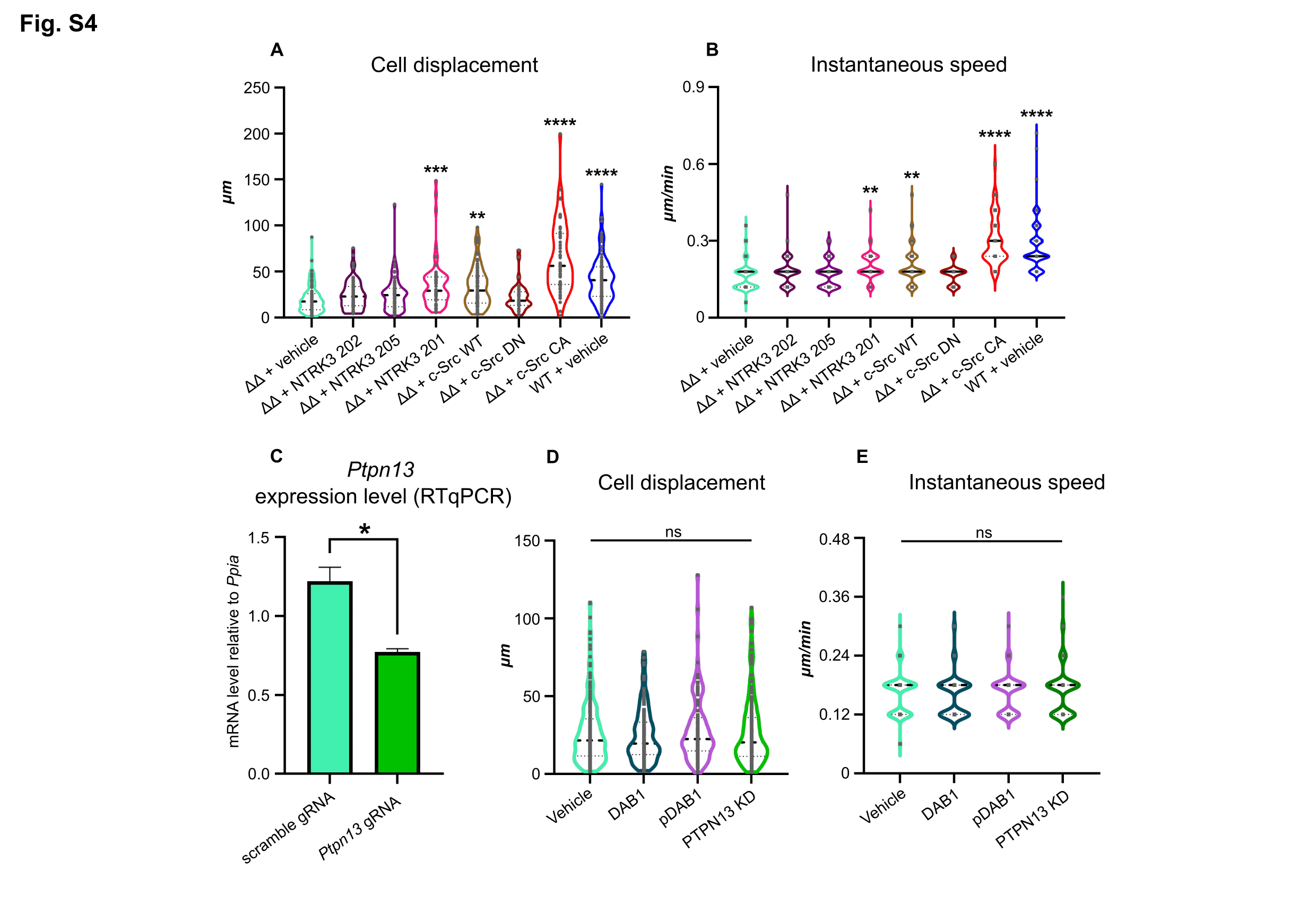

Supplement: Supplementary file 9 — Figure S4 Mobility rescue assays. [file 41420_2023_1615_MOESM9_ESM.png]
