# Supplementary material for: The Neurod1/4-Ntrk3-Src pathway regulates gonadotrope cell adhesion and motility
Source: Cell Death Discov. 2023 Sep 1;9:327. doi: 10.1038/s41420-023-01615-7 (PMC10474047; doi:10.1038/s41420-023-01615-7)

Blot related to the figure 5

Exposition time : 2s

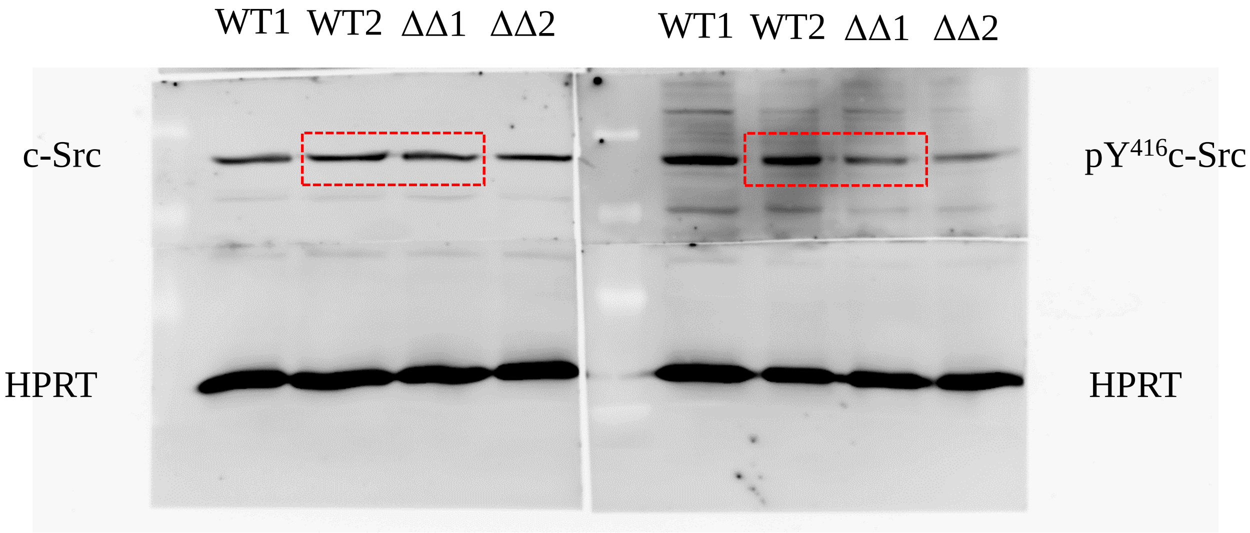

Exposition time : 1.2s

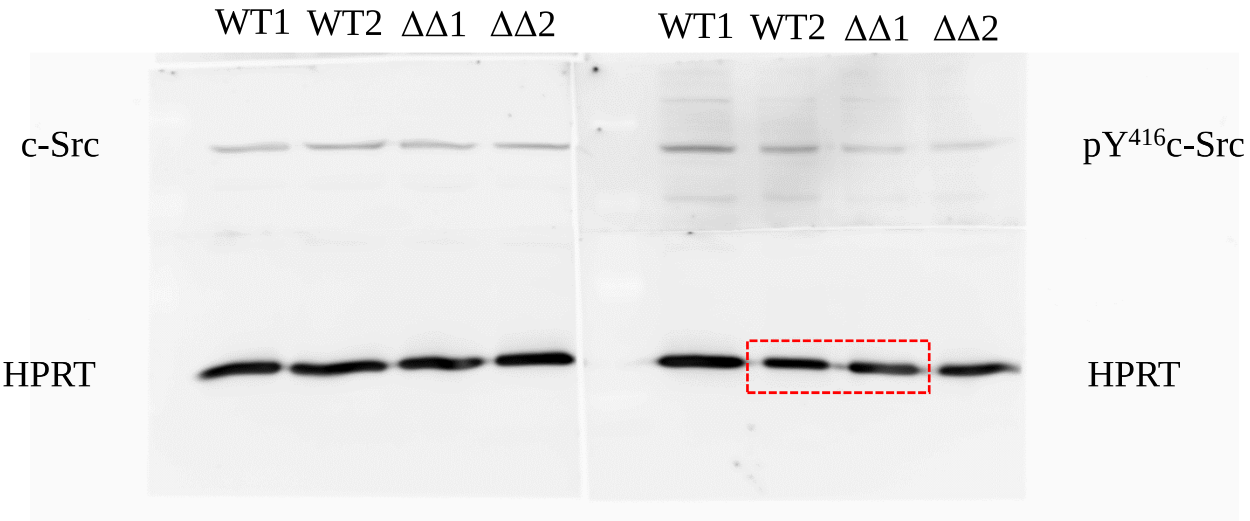

Blot related to the figure 6

Exposition time : 1s

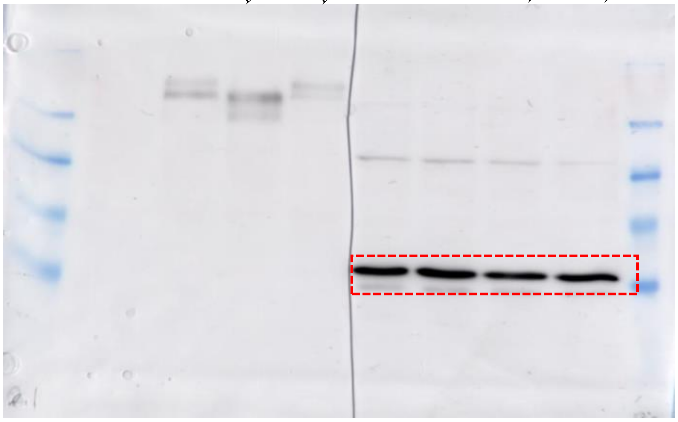

Exposition time : 2s

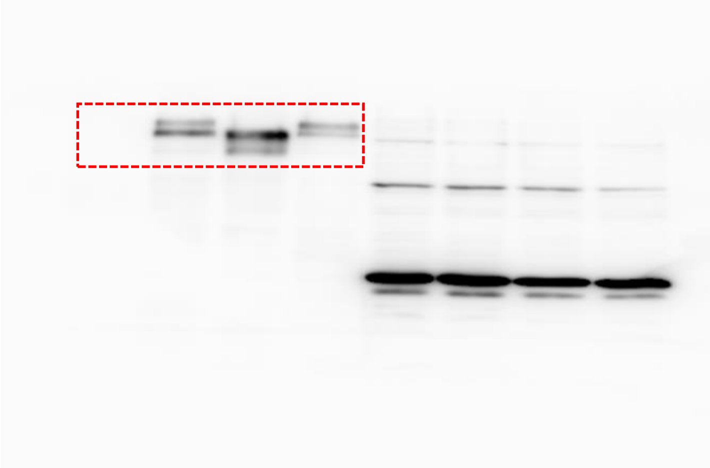

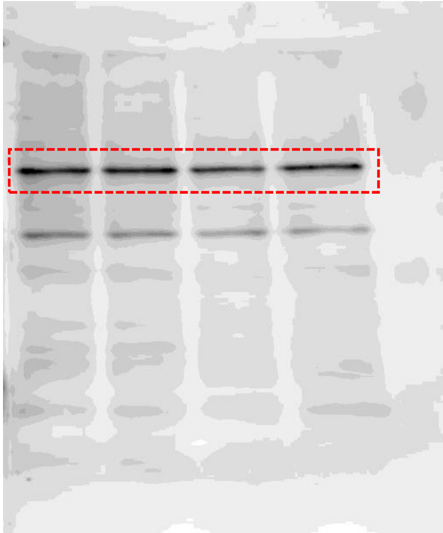

Blot related to the figure 6

Input

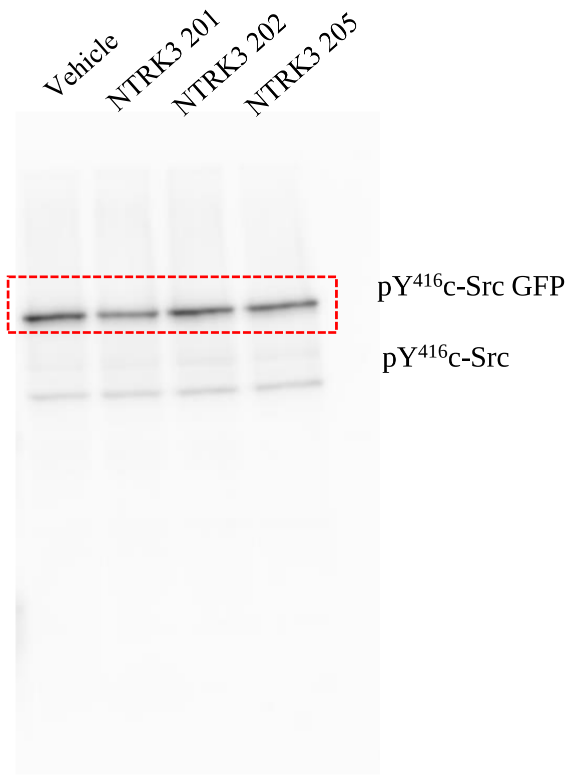

IP

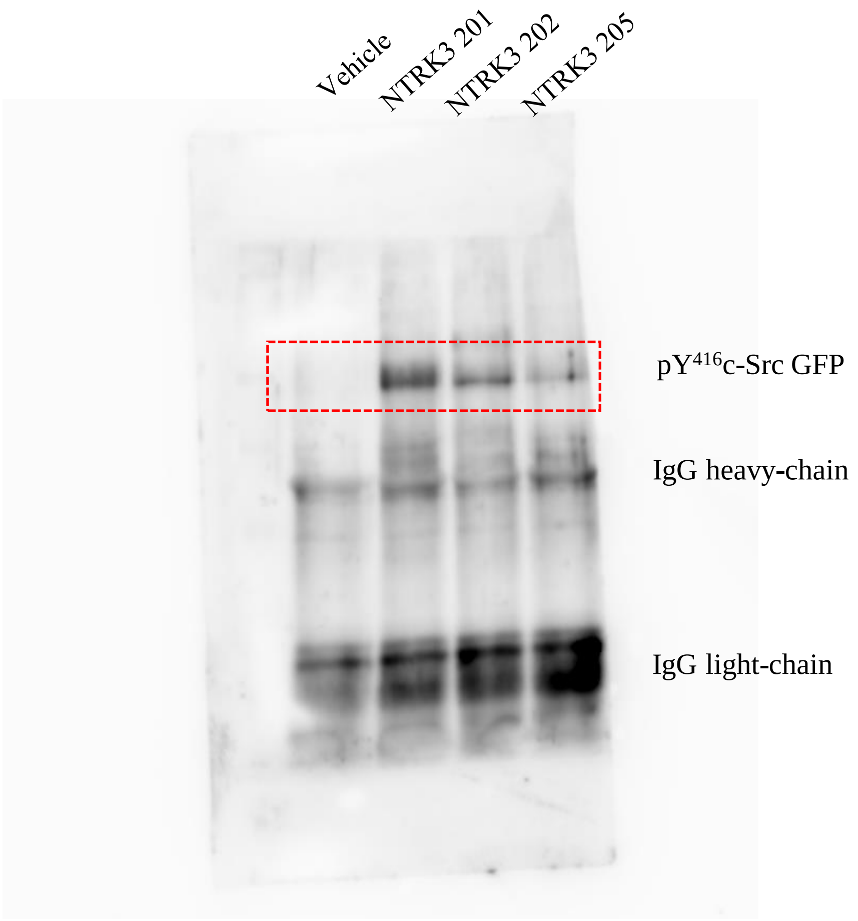

Blot related to the figure 6

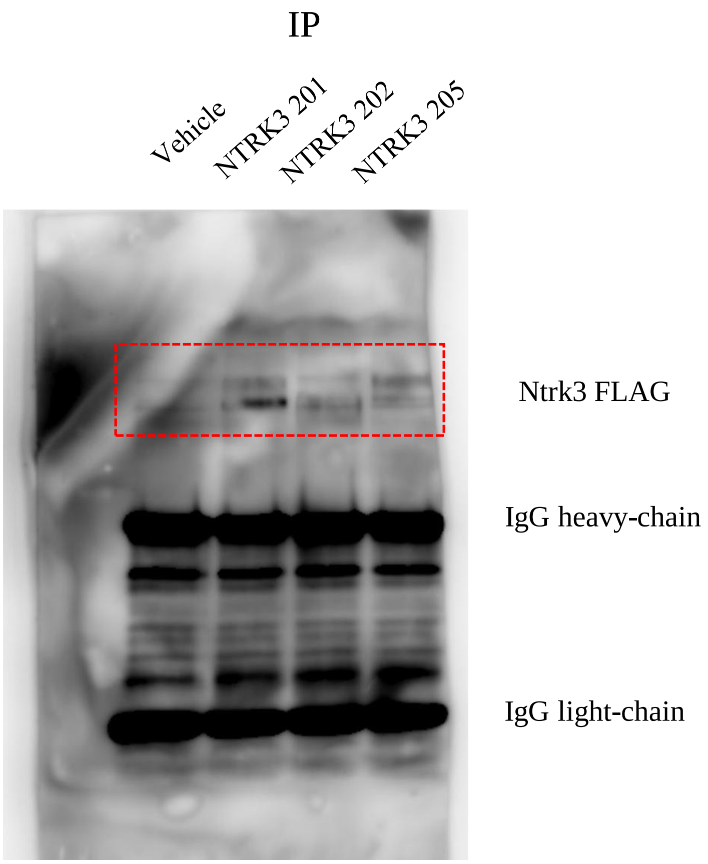

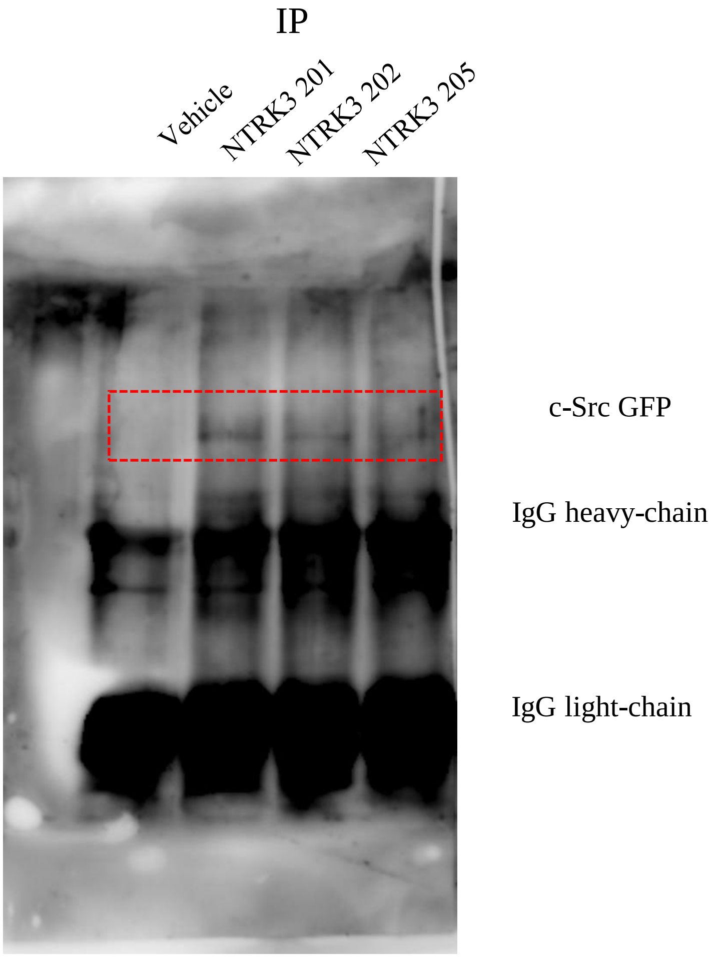

Blot related to the figure S3

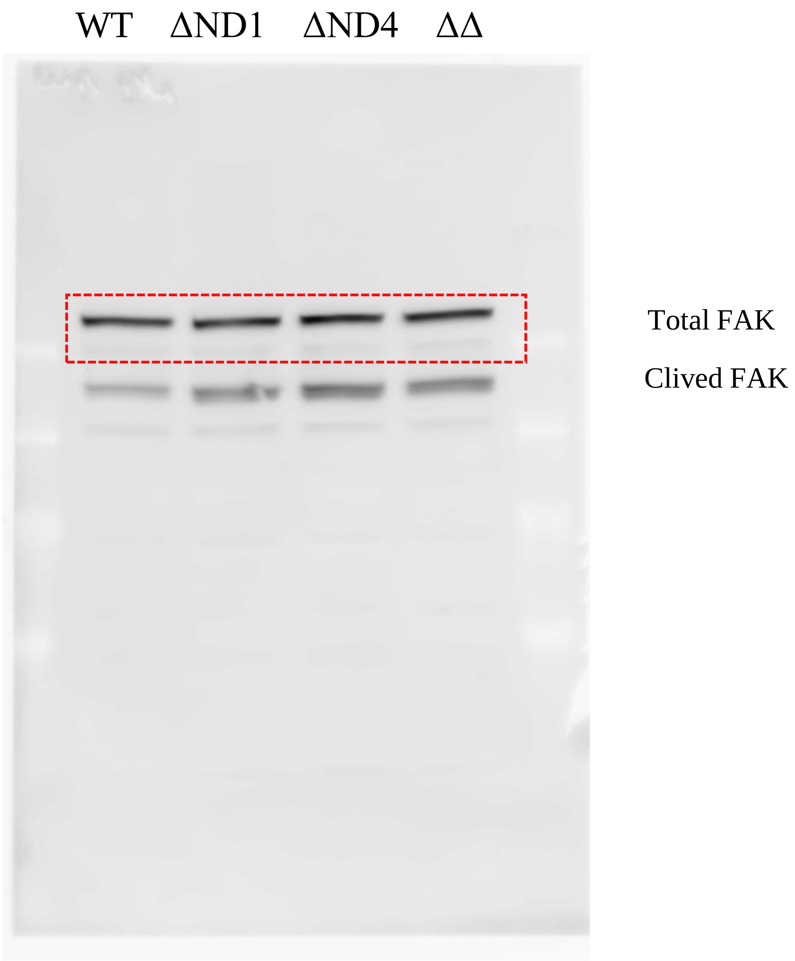

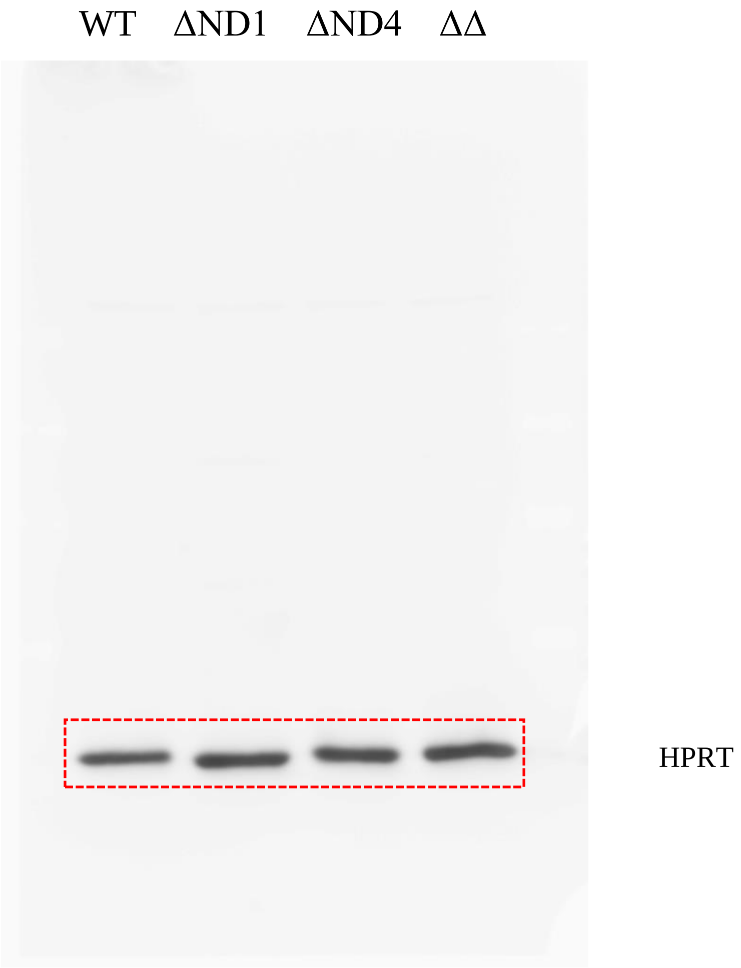

Supplement: Supplementary file 10 — Uncropped Western Blots [file 41420_2023_1615_MOESM10_ESM.pdf]
