# Supplementary material for: The Neurod1/4-Ntrk3-Src pathway regulates gonadotrope cell adhesion and motility
Source: Cell Death Discov. 2023 Sep 1;9:327. doi: 10.1038/s41420-023-01615-7 (PMC10474047; doi:10.1038/s41420-023-01615-7)
Supplement: Supplementary file 6 [file 41420_2023_1615_MOESM1_ESM.odt]

Supplementary Information

Movie M1 Time lapse video microscopy of motility experiment of αT3-1 WT cells

Movie M2 Time lapse video  lapse microscopy of motility experiment of αT3-1 ΔΔ cells

Uncropped Western Blots

Supplementary Table 1 ND1/4 potential target gene in immature gonadotrope pathway analysis

Supplementary Table 2 Primers and antibodies

Figure S1 FluoroBlok transwell migration assay.

WT, ΔND1 and ΔND4 cells were seeded in FluoroBlok transwell inserts and treated with FBS (10% in DMEM). The Relative Fluorescence Unit is the ratio of post-migratory fluorescence of FBS-treated over untreated cells. Data are the normalized mean ± SEM of 6 independent experiments. Migration of ΔND1, ΔND4 and ΔΔ cells was compared to WT by ANOVA followed by Dunnett’s multiple comparison test. ** p < 0.01, ns: not significant.

Figure S2 Subcellular localization of total Cortactin in WT and ΔΔ cells.

Representative confocal images of Cortactin-labeled WT and ΔΔ cells and fluorescence quantification. Cortactin was immuno-localized on fixed cells labeled with phalloidine-TRITC and DAPI (Green: Cortactin, Red: F-actin, Blue: Nuclei). Bar graph of the mean signal fluorescence intensity per cell quantified on 15 different images for each genotype. Mean cell intensity was compared between ΔΔ and WT cells. ns: not significant (Mann-Whitney test).

Figure S3 FAK expression in WT and ΔΔ cells. 

A) Ptk2 (Fak) gene expression levels were measured by RT-qPCR and normalized to Ppia. Data are the normalized mean ± SEM of three independent experiments. Ptk2 gene expression in ΔND1, ΔND4, ΔΔ cells were compared to WT by ANOVA followed by Dunnett’s multiple comparison test. ns: not significant.

B) Western blot analysis of total FAK in WT, ΔND4, ΔND1 and ΔΔ cells. HPRT was used as loading control.

Figure S4 Mobility rescue assays

A-B) Cell motility rescue assay. ΔΔ cells were co-transfected with H2B-mCherry and either with GFP (vehicle), Ntrk3-201, Ntrk3-202, Ntrk3-205, c-Src DN, c-Src WT or c-Src CA expression plasmids. WT cells, transfected with GFP expression plasmids (Vehicle), were used as a positive control. A) Total cell displacement analysis. The level of cell displacement (in µm) was analysed using ImageJ TrackMate. Mean displacement of transfected ΔΔ cells and GFP transfected WT cells (WT + Vehicle) was compared to GFP transfected ΔΔ cells (ΔΔ + vehicle) by ANOVA followed by Dunnett’s multiple comparison test. ** p < 0.01, *** p < 0.001 and **** p < 0.0001. B) Cell velocity analysis. Cells instantaneous speed (µm/minute) was calculated from videomicroscopy analysis of transfected cells over 16 hours, using ImageJ TrackMate. Mean cell velocity of transfected ΔΔ cells and GFP transfected WT (WT + Vehicle) was compared to GFP transfected ΔΔ cells (ΔΔ + vehicle) by ANOVA followed by Dunnett’s multiple comparison test. ** p < 0.01 and **** p < 0.0001.

C) Ptpn13 knock-down in ΔΔ cells by CRISPRi. ΔΔ cells were co-transfected with a KRAB-dCas9-GFP along with untargeting gRNA (scramble gRNA) or gRNA targeting Ptpn13 proximal promoter (Ptpn13 gRNA). Ptpn13 gene expression levels were measured by RT-qPCR and normalized to Ppia. Data are the normalized mean ± SEM of three independent experiments. Ptpn13 KD efficiency was tested using a Student’s t test. * p < 0.05

D-E) Cell motility rescue assay. ΔΔ cells were co-transfected with H2B-mCherry and DAB1 2A-GFP expression plasmids (WT or phospho-mimetic mutant) or with CRISPRi-Ptpn13 dCas9-GFP (PTPN13 KD). Cells were seeded on Matrigel-coated culture surface until attachment and motility of GFP-positive cells was tracked using live video time-lapse microscopy over 16 hours. Tracks were analysed using ImageJ TrackMate. D) Cell displacement analysis. The level of cell displacement (in µm) was analysed using ImageJ TrackMate. Mean displacement of overexpressing DAB1 or KD Ptpn13 ΔΔ cells was compared to GFP transfected ΔΔ cells (vehicle) by ANOVA followed by Dunnett’s multiple comparison test. ns = not significant. E) Cell velocity analysis. Cells instantaneous speed (µm/minute) was calculated from the videomicroscopy analysis of transfected ΔΔ cells over 16 hours, using ImageJ TrackMate. Mean velocity of overexpressing DAB1 or KD Ptpn13 ΔΔ cell was compared to GFP transfected ΔΔ cells (vehicle) by ANOVA followed by Dunnett’s multiple comparison test. ns = not significant.
